# PPP2R3C serves as a negative regulator associated with reduced T cell hyperactivation and renal protection in lupus

**DOI:** 10.1002/ctm2.70716

**Published:** 2026-06-15

**Authors:** Xuan Fang, Yi Qin, Jinhui Tao, Zhou Zhou, Minglong Cai, Hong Zhang, Xiangpei Li, Xiaomei Li, Zhu Chen

**Affiliations:** ^1^ Department of Rheumatology and Immunology The First Affiliated Hospital of University of Science and Technology of China Hefei Anhui China; ^2^ Department of Laboratory Medicine The First Affiliated Hospital of University of Science and Technology of China Hefei Anhui China; ^3^ Department of Internal Medicine 3‐Rheumatology and Immunology Friedrich‐Alexander‐University (FAU) Erlangen‐Nürnberg and Universitätsklinikum Erlangen Erlangen Germany; ^4^ Department of Allergy and Clinical Immunity The First Affiliated Hospital of University of Science and Technology of China Hefei Anhui China; ^5^ Department of Nephrology The First Affiliated Hospital of University of Science and Technology of China Hefei Anhui China; ^6^ Department of Dermatology The First Affiliated Hospital of University of Science and Technology of China Hefei Anhui China

**Keywords:** gene therapy, PPP2R3C, systemic lupus erythematosus, T cell hyperactivation

## Abstract

**Background:**

CD4^+^ T cell hyperactivation is a pivotal driver of systemic autoimmunity in systemic lupus erythematosus (SLE), yet the molecular regulators that set its activation threshold remain poorly defined.

**Methods:**

PPP2R3C expression was measured by qRT‐PCR and Western blotting in CD4^+^ T cells from 45 SLE patients and 37 healthy controls, as well as in pristane‐induced lupus (PIL) mice. Jurkat cells with PPP2R3C knockdown or overexpression were generated by lentiviral transduction. Signaling mechanisms were dissected using transcriptome sequencing and calcium flux assays. In vivo, PPP2R3C was restored in PIL mice via T cell‐targeted Ark313 vector or systemic AAV9 delivery; disease progression was assessed at 24 and 48 weeks after pristane induction.

**Results:**

We identified the protein phosphatase 2A regulatory subunit PPP2R3C as a critical and selective negative regulator of T cell receptor (TCR) signaling, which was downregulated in CD4^+^ T cells from SLE patients and pristane‐induced lupus (PIL) mice. Reduced PPP2R3C expression was also observed in the kidneys of both PIL mice and lupus nephritis patients. In PIL mice, this reduction was evident in podocytes, endothelial cells, and mesangial cells, indicating widespread downregulation across kidney resident cell populations. Mechanistically, PPP2R3C deficiency enhanced T cell activation, cytokine production, and calcium flux by potentiating PLCγ1 phosphorylation and subsequent TCR‐driven JNK/c‐Jun signaling, whereas its overexpression produced opposing effects. To assess therapeutic potential, we restored PPP2R3C expression in PIL mice using two complementary gene delivery strategies. T cell‐targeted reconstitution via the Ark313 vector potently suppressed T cell activation and autoantibody production at an early stage (24 weeks), culminating in markedly attenuated proteinuria and glomerular immune complex deposition by late‐stage disease (48 weeks). Systemic delivery of AAV9‐PPP2R3C provided comprehensive therapeutic effects, ameliorating renal pathology while also normalizing immune dysregulation in both the thymus and spleen across both time points.

**Conclusions:**

Our findings identify PPP2R3C as an important contributor to SLE pathogenesis and support its restoration as a gene therapy approach worthy of further exploration for its ability to rebalance immunity and mitigate tissue injury in SLE.

**Key points:**

- PPP2R3C is selectively downregulated in SLE CD4^+^ T cells and correlates with disease activity.
- It restrains the PLCγ1‐JNK axis to limit T cell hyperactivation and cytokine production.
- Gene therapy restoring PPP2R3C suppresses autoimmunity and lupus nephritis in mice, and its reduction in kidney resident cells indicates a direct renoprotective effect, positioning PPP2R3C as a promising therapeutic target for SLE.

## Graphical abstract

Downregulation of PPP2R3C in lupus CD4^+^ T cells unleashes JNK/c‑Jun and PLCγ1 signalling, driving calcium influx and inflammatory cytokine production. Reconstitution of PPP2R3C expression curbs T cell activation, lowers autoantibody levels and alleviates lupus nephritis, highlighting PPP2R3C as a promising therapeutic candidate.

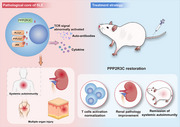

## 1 INTRODUCTION

Systemic lupus erythematosus (SLE) represents a systemic autoimmune disease featuring both impaired immune self‐tolerance and sustained inflammation.^1^ A central driver of SLE pathogenesis is CD4^+^ T cell hyperactivation, which arises from lowered T cell receptor (TCR) activation thresholds and impaired peripheral tolerance.^2^ TCR engagement with major histocompatibility complex (MHC)–antigen complexes on antigen‐presenting cells (APCs) triggers signalosome assembly, activating downstream pathways including NF‐κB, JNK, ERK, NFAT and mTOR.^3^ These cascades regulate T cell proliferation, cytokine production and tissue infiltration, ultimately perpetuating multi‐organ injury.^2^

In eukaryotic cells, protein phosphatase 2A (PP2A) serves as a major serine/threonine phosphatase, consisting of a catalytic subunit (PP2Ac), a structural subunit (PP2Aa) and various regulatory subunits. The catalytic subunit is responsible for driving phosphatase activity, while the regulatory subunits affect both substrate specificity and the subcellular localisation of the PP2A holoenzyme.^4^, ^5^ Accumulating evidence indicates that PP2A plays an essential role in T cell development, function and the regulation of downstream signalling pathways triggered by TCR activation.^6^, ^7^ Depletion of the PP2A regulatory subunit PPP2R5C has been demonstrated to increase the phosphorylation levels of IκB kinase (IKK) and IκBα, resulting in enhanced NF‐κB activity following TCR stimulation.^8^ Similarly, inhibition of PPP2R2D has been found to enhance the proliferation, cytokine production and cytotoxic capacity of CD4^+^ and CD8^+^ T cells.^9^

In the context of SLE, the Bβ regulatory subunit PPP2R2B, originally considered brain‐specific, is expressed in T cells and is essential for IL‐2 deprivation‐induced apoptosis; its defective upregulation in approximately 50% of SLE patients contributes to the survival of autoreactive T cells.^10^ Furthermore, PPP2R2B expression is suppressed in autoimmune T cells through inflammation‐driven promoter hypermethylation, which prevents CCCTC‐binding factor (CTCF) from binding to its target region.^11^ Conversely, SLE‑derived T cells exhibit higher levels of the regulatory subunit PPP2R2A, which then facilitates Th1 and Th17 cell differentiation through the GEF‑H1/RhoA/ROCK signalling cascade.^12^ Further recent findings have shown that PPP2R2A modulates NAD^+^ biosynthesis via the nicotinamide riboside‑mediated salvage pathway to regulate the differentiation of T cell subsets in the context of systemic autoimmunity.^13^ Collectively, these findings reveal that different PP2A regulatory subunits exhibit distinct and even opposing expression patterns in SLE. This heterogeneity underscores the complexity of PP2A holoenzyme regulation in autoimmunity, suggesting that each regulatory subunit may contribute to a unique aspect of T cell dysfunction.

Besides impacting T cell activation, PP2Ac plays a critical role in the development and function of Tregs. Silencing PP2Ac in Tregs augments mTORC1 signalling, thereby compromising their immunosuppressive function and triggering the production of IgG autoantibodies against 76 autoantigens, including both tissue‐restricted and lupus‐associated nuclear antigens. This loss of immune tolerance culminated in the development of severe autoimmune disease.^14^ Furthermore, the regulatory subunit PPP2R3C influences thymocyte development and the survival of CD4 and CD8 double‐positive (DP) cells by inhibiting JNK‐mediated apoptosis signalling.^15^ The role of PP2A is multifaceted and context‑dependent. Although its significance in T cell biology is well‐established, the precise contributions of its various regulatory subunits to autoimmune diseases remain poorly defined.

The present study demonstrated significantly lower PPP2R3C expression in CD4+ T cells of SLE patients and pristane‐induced lupus (PIL) mice, as well as in kidney tissues of PIL mice and lupus nephritis (LN) patients. This downregulation correlated with enhanced TCR‑proximal signalling, including increased PLCγ1 phosphorylation and JNK‑c‐Jun activation, leading to T cell hyperactivation and proinflammatory cytokine release. In vivo, using two distinct delivery approaches: systemic AAV9 and T cell‐targeted Ark313 engineered AAV, we demonstrated that PPP2R3C augmentation effectively suppressed T cell activation and systemic autoimmunity at both early (24‐week) and late (48‐week) disease stages. Notably, the therapeutic outcomes were influenced by the expression pattern: Systemic AAV9 delivery conferred comprehensive protection, resolving immune dysregulation and mitigating organ damage as early as 24 weeks, while T cell‐restricted Ark313 editing selectively moderated immune activation, with renal protection manifesting in the later stages of the disease. These findings establish the restoration of PPP2R3C as a promising strategy for the treatment of SLE and highlighting the importance of both immunomodulation and direct renal protection.

## 2 RESULTS

### 2.1 Reduced expression of PPP2R3C in SLE CD4+ T cells correlates with disease progression

**FIGURE 1:**
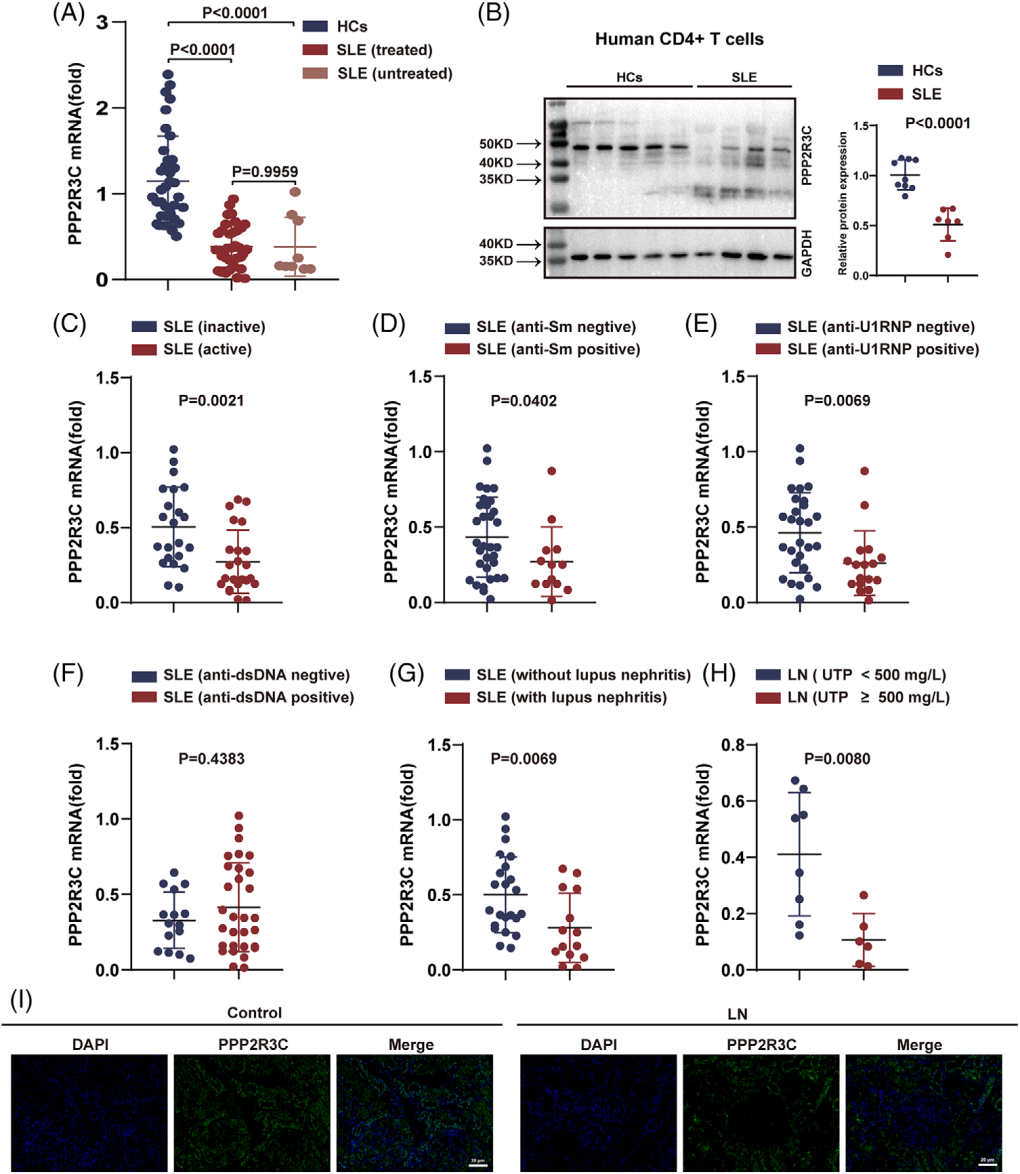
Downregulation of PPP2R3C in systemic lupus erythematosus (SLE) CD4^+^ T cells associated with SLE disease progression. (A) Relative PPP2R3C mRNA levels in CD4^+^ T cells from healthy controls (HCs; *n* = 37) and SLE patients (*n* = 45), subdivided into drug‐naive (*n* = 9) and previously treated cohorts (*n* = 36). (B) Western blot analysis of PPP2R3C protein expression in CD4^+^ T cells of SLE (*n* = 7) and HCs (*n* = 9). (C) PPP2R3C mRNA levels in CD4^+^ T cells from SLE patients with active (SLEDAI ≥6, *n* = 23) or inactive (SLEDAI <6, *n* = 22) disease. (D, E) PPP2R3C mRNA levels in CD4^+^ T cells from SLE patients stratified by seropositivity for anti‑Sm‑negative (*n* = 32) and anti‑Sm‑positive (*n* = 13) (D) and anti‐nRNP/Sm‑negative (*n* = 28) and anti‐nRNP/Sm‑positive (*n* = 17) (E) antibodies. (F) PPP2R3C mRNA levels in CD4^+^ T cells from SLE patients stratified by seropositivity for anti‐dsDNA‑negative (*n* = 15) and anti‐dsDNA‑positive (*n* = 30) antibodies. (G) PPP2R3C mRNA expression in SLE patients with (*n* = 14) and without (*n* = 23) lupus nephritis (LN). (H) PPP2R3C mRNA expression in LN patients stratified by urinary total protein (UTP) levels (<500 mg/L, *n* = 8; ≥500 mg/L, *n* = 6). (I) Immunofluorescence staining of PPP2R3C in kidney biopsies from lupus nephritis patients and disease controls (*n* = 3). Data are presented as mean ± SD. For qPCR data, each dot represents an individual patient, and each sample was measured in technical triplicate; For multi‐group comparisons in panel (A), one‐way ANOVA followed by Tukey's multiple comparisons test was used. For two‐group comparisons in panels (B–H), the Mann–Whitney *U*‐test (two‐tailed) was applied.

In order to clarify the connection between PP2A and SLE, we performed an examination of the mRNA expression levels of essential PP2A subunits, which included the structural subunit PPP2R1A, the catalytic subunit PPP2CA and various regulatory components (PPP2R2A/D, PPP2R3A/B/C, PPP2R5A/B/C/D/E) in CD4+ T cells taken from both SLE patients and healthy controls (HCs). Notably, transcripts of PPP2R3C showed a selective downregulation in CD4+ T cells from SLE patients when compared to HCs, while the expression of other subunits did not change (Figure S1A). Considering the possible effects of previous medication, we classified the patients into two categories: nine drug‑naive cases with a first episode and 36 cases who had undergone prior drug treatment. A significant downregulation of PPP2R3C mRNA was found in SLE patients versus HCs, irrespective of treatment history (Figure 1A). Consistently, PPP2R3C protein levels were reduced in SLE CD4^+^ T cells (Figure 1B), However, PPP2R3C had relatively similar expression levels observed in CD8^+^ T or CD19^+^ B cells of SLE and HCs (Figure S1B,C), indicating CD4^+^ T‐cell‐specific dysregulation of PPP2R3C may be involved in SLE progression. To investigate the correlation between the PPP2R3C in CD4^+^ T cells and clinic manifestation in SLE, these patients with SLE were categorised into active (SLEDAI ≥6) and inactive (SLEDAI <6) phases. The levels of PPP2R3C mRNA were significantly decreased in active patients compared to inactive patients (Figure 1C). Furthermore, PPP2R3C mRNA levels were significantly lower in patients who tested positive for anti‐Sm and anti‐nRNP/Sm antibodies compared to those who were negative for these antibodies (Figure 1D,E). In contrast, no significant difference in PPP2R3C expression was observed between patients who were positive or negative for anti‐dsDNA (Figure 1F), anti‐nucleosome antibodies (ANuA) and anti‐C1q antibodies (Figure S1D). We further examined the association between PPP2R3C expression and LN. Among the SLE patients with available urinary protein data (*n* = 37), PPP2R3C mRNA levels were significantly lower in patients diagnosed with LN (*n* = 14) compared to those without nephritis (*n* = 23; Figure 1G). Moreover, among patients with documented proteinuria, those with urinary total protein (UTP) ≥500 mg/L (*n* = 6) exhibited significantly lower PPP2R3C expression compared to those with UTP <500 mg/L (*n* = 8; Figure 1H). To further validate the clinical relevance of our findings, we examined PPP2R3C expression in kidney biopsies from three LN patients and three disease controls (clinical information is provided in Table S2). Immunofluorescence analysis revealed that PPP2R3C protein expression was significantly reduced in the kidney tissues of LN patients compared to controls. Quantitative analysis is provided in Figure S1E. Additionally, PPP2R3C mRNA expression showed no significant correlation with platelet (PLT) count, white blood cell (WBC) count, complement C3, or C4 levels (Figure S1F). These findings nominate PPP2R3C as a PP2A subunit with potential immunopathological relevance to SLE.

### 2.2 PPP2R3C deficiency potentiates TCR signalling and effector functions

**FIGURE 2:**
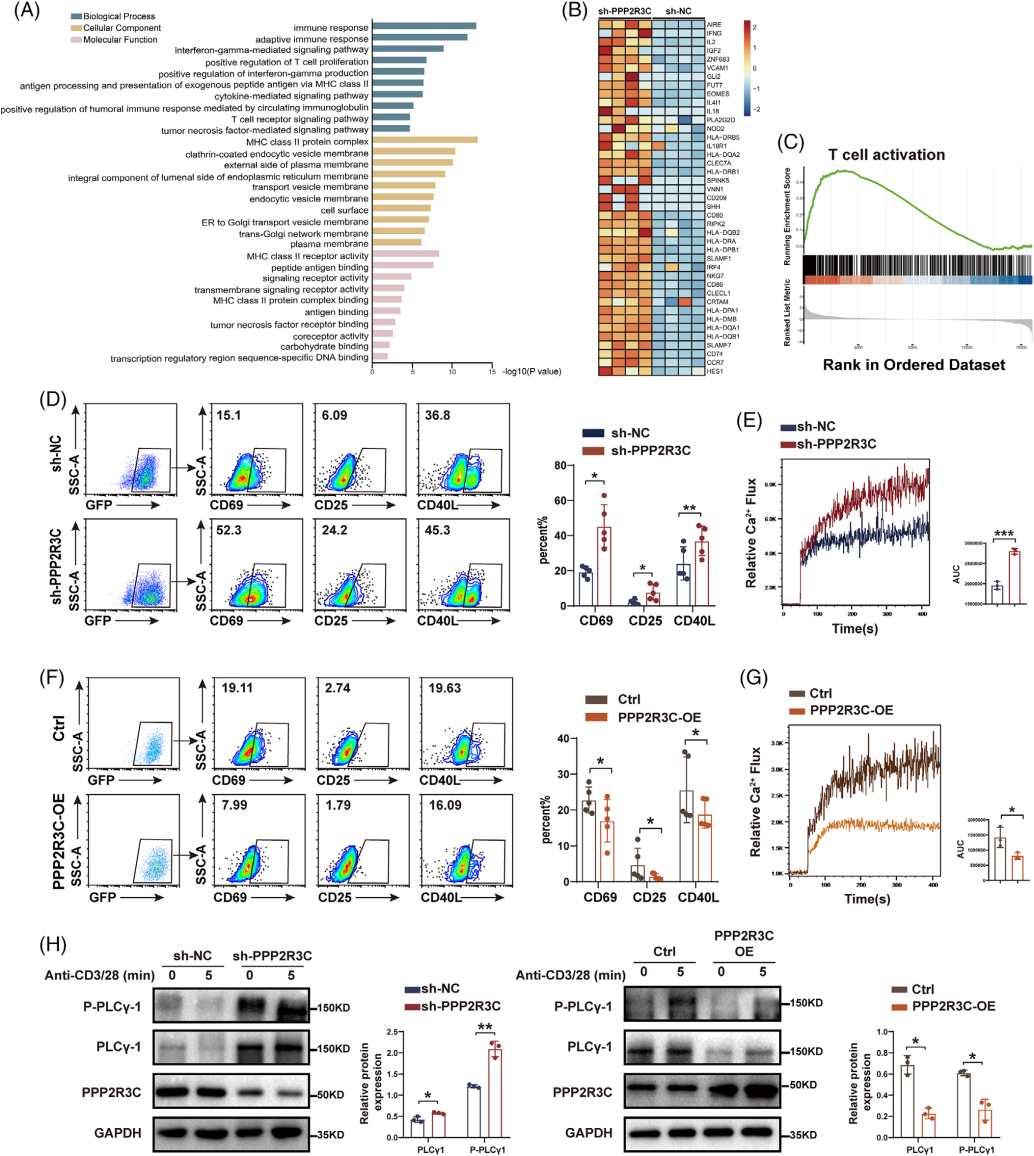
PPP2R3C deficiency potentiates T cell receptor (TCR) signalling and effector functions. (A) GO enrichment analysis of biological processes. Cellular Component and molecular functions significantly altered upon PPP2R3C knockdown (sh‐PPP2R3C) compared to control (sh‐NC; *n* = 4). (B) Heat maps depict PPP2R3C‐regulated genes linked to T cell activation. (C) GSEA analysis of T cell activation pathway enrichment in sh‐PPP2R3C Jurkat cells. (D) Representative flow cytometry plots (left) and quantification (right) of the activation markers CD69, CD25 and CD40L on sh‑NC and sh‑PPP2R3C Jurkat cells following stimulation with anti‑CD3 (5 µg/mL) and anti‑CD28 (2 µg/mL) for 24 h (*n* = 5 independent experiments). Each experiment consisted of paired control (sh‑NC) and PPP2R3C‑knockdown (sh‑PPP2R3C) cells derived from the same batch of Jurkat cells. (E) Intracellular Ca^2^^+^ flux in sh‐NC and sh‐PPP2R3C cells following TCR/CD28 stimulation, measured by flow cytometry using Rhod‐2/AM labelling (*n* = 3). (F) Expression of CD69, CD25 and CD40L on control and PPP2R3C‐OE Jurkat cells following anti‐CD3 (5 µg/mL) and anti‐CD28 antibodies (2 µg/mL) for 24 h stimulation (*n* = 5). (G) Intracellular Ca^2^^+^ flux in control and PPP2R3C‐OE cells following TCR/CD28 stimulation. Representative data were collected and expressed as mean ± SD from three independent experiments (*n* = 3). (H) Immunoblot analysis of total and phosphorylated PLCγ1 (Tyr783) in Jurkat T cells with PPP2R3C knockdown or overexpression following anti‐CD3 (5 µg/mL) and anti‐CD28 antibodies (2 µg/mL) for 5 min stimulation (*n* = 3). Data are from a single experiment representative of at least two independent experiments. Data are presented as mean  ±  SD. *p* values were calculated by paired two‑tailed Student's *t*‑test (* indicates *p* < .05, ** indicates *p* < .01, *** indicates *p* < .001, ns, not significant).

To interrogate the functional role of PPP2R3C in T cell biology, we employed the Jurkat T cells with PPP2R3C knockdown or overexpression via Lentivirus (Figure S2A). RNA sequencing was used to investigate alterations in gene expression of shRNA‐PPP2R3C (sh‐PPP2R3C) versus shRNA‐NC (sh‐NC) Jurkat T cells, with 109 genes upregulated and 14 genes downregulated in sh‐PPP2R3C compared to sh‐NC (Figure S2B). To explore functional pathways related to PPP2R3C, we conducted a gene ontology (GO) enrichment analysis. Figure 2A demonstrates that transcripts associated with PPP2R3C reduction were significantly enriched in immune‑related pathways, for example, positive regulation of T cell proliferation and the interferon‑gamma‑mediated signalling pathway. Additionally, we observed enrichment in the molecular function category for MHC class II receptor activity. This immunostimulatory phenotype was corroborated by gene set enrichment analysis (GSEA), which demonstrated heightened activity of the T cell activation pathway and elevated expression of 41 key genes, including IL‐2 and IFNG, in sh‐PPP2R3C cells (Figure 2B,C). The finding was confirmed by the enhanced surface expression of activation markers CD69, CD25 and CD40L (Figure 2D) and amplified IL‐2/IFN‐γ secretion at both protein and transcriptional levels in sh‐PPP2R3C cells (Figure S2C) versus sh‐NC under TCR/CD28 co‐stimulation. Intracellular Ca^2^^+^ flux was also augmented in sh‐PPP2R3C cells (Figure 2E). Conversely, PPP2R3C overexpression (PPP2R3C‐OE) cells exhibited suppressed activation markers (Figure 2F) and diminished production of IL‐2 and IFN‐γ (Figure S2D), along with attenuated Ca^2^^+^ responses (Figure 2G) under TCR/CD28 co‐stimulation. These findings indicate that PPP2R3C may regulate TCR signalling activation.

Following TCR engagement, T cells drive the recruitment and activation of PLCγ1, which in turn breaks down PIP2 to yield IP3 and DAG as second messengers, culminating in sustained Ca^2^^+^ influx. This elevation in intracellular Ca^2^^+^ is essential for the activation of the Ca^2^^+^–calcineurin–NFAT signalling pathway, which drives cytokine gene expression and T cell effector functions.^16^ Given that PPP2R3C knockdown markedly augmented TCR‑induced Ca^2^^+^ flux, we next examined whether PPP2R3C acts upstream of this process by modulating PLCγ1 signalling. Consistent with this hypothesis, PPP2R3C deficiency significantly increased total and phosphorylated PLCγ1 levels, while PPP2R3C overexpression had the opposite effect (Figure 2H), positioning PLCγ1 as a key target for PPP2R3C‑mediated regulation of Ca^2^^+^‑dependent T cell activation.

### 2.3 PPP2R3C restricts T cell activation by targeting JNK pathway

**FIGURE 3:**
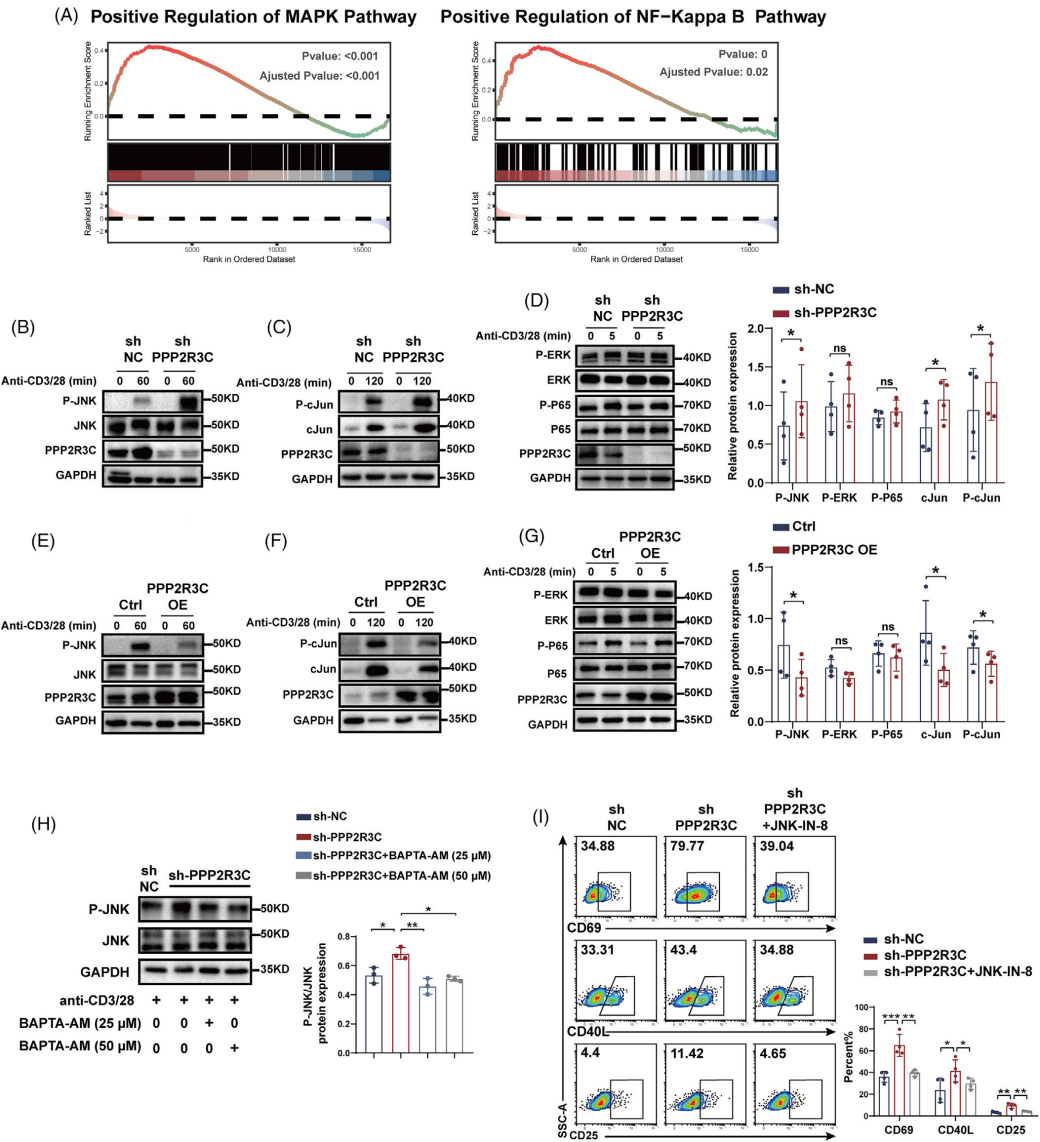
PPP2R3C restricts T cell activation by targeting JNK pathway. (A) GSEA of RNA‐sequencing data from sh‐PPP2R3C versus sh‐NC Jurkat cells, showing enrichment for MAPK and NF‐κB signalling pathways. (B, C) Immunoblots (left) and quantification (right) of phosphorylated JNK (B), phosphorylated c‐Jun and total c‐Jun (C) in serum‐starved sh‐NC and sh‐PPP2R3C Jurkat cells at indicated time points after anti‐CD3 (5 µg/mL) and anti‐CD28 antibodies (2 µg/mL) stimulation (*n* = 4). (D) Immunoblots for phosphorylated and total ERK and p65 in sh‐NC and sh‐PPP2R3C cells under anti‐CD3 (5 µg/mL) and anti‐CD28 antibodies (2 µg/mL) stimulation (*n* = 4). (E, F) Immunoblots (left) and quantification (right) of phosphorylated JNK (E), phosphorylated c‐Jun and total c‐Jun (F) in serum‐starved control and PPP2R3C‐OE Jurkat cells at indicated time points after anti‐CD3 (5 µg/mL) and anti‐CD28 antibodies (2 µg/mL) stimulation (*n* = 4). (G) Immunoblots for phosphorylated ERK and p65 in control and PPP2R3C‐OE cells underanti‐CD3 (5 µg/mL) and anti‐CD28 antibodies (2 µg/mL) stimulation (*n* = 4). (H) Immunoblot analysis of P‐JNK in PPP2R3C‑knockdown Jurkat T cells treated with or without BAPTA‑AM (25 µM or 50 µM) followed by anti‑CD3/CD28 stimulation for 60 min (*n* = 3). (I) Surface expression of CD69, CD25 and CD40L on sh‐PPP2R3C Jurkat cells treated with or without the JNK inhibitor JNK‐IN‐8 following anti‑CD3/CD28 stimulation (*n* = 4). Data are from a single experiment representative of at least two independent experiments. Data are presented as mean  ±  SD. *p* values were calculated by paired two‑tailed Student's *t*‑test (* indicates *p* < .05, ** indicates *p* < .01, *** indicates *p* < .001, ns, not significant).

TCR/CD28 stimulation triggers multiple common downstream signalling pathways, including the ERK, JNK, NF‐κB, AKT and mTOR signalling pathways.^17^ RNA‐sequencing analysis implicated MAPK and NF‐κB signalling enrichment in sh‐PPP2R3C T cells (Figure 3A). To investigate which signalling pathways were affected by PPP2R3C, we deprived transfected Jurkat cells of nutrients overnight in a serum‐free environment and subsequently exposed the cells to TCR/CD28 stimulation at different time points. We found that deficiency of PPP2R3C triggered JNK activation, determined by hyperphosphorylation of JNK at Thr183/Tyr185 (Figure 3B). Concomitantly, total and phosphorylated c‐Jun (Ser73) were elevated (Figure 3C). Conversely, PPP2R3C‐OE cells exhibited blunted phosphorylation of JNK (Figure 3E), in company with reduced total and phosphorylated c‐Jun (Ser73; Figure 3F). Notably, ERK and NF‐κB signalling remained unaffected (Figure 3D,G), indicating that PPP2R3C selectively constrains JNK signalling rather than broadly suppressing TCR‐driven cascades.

Having established that PPP2R3C regulates PLCγ1 phosphorylation and calcium flux, we next asked whether calcium is required for the enhanced JNK phosphorylation observed in PPP2R3C‑deficient cells. Pretreatment with the intracellular calcium chelator BAPTA‑AM (25 µM and 50 µM) completely abolished the increased JNK phosphorylation in sh‑PPP2R3C cells, bringing it to levels comparable to control cells (Figure 3H). These results demonstrate that calcium is required for JNK activation in PPP2R3C‑deficient T cells.

To confirm the centrality of JNK involved in sh‐PPP3R3C T cell activation, we treated sh‐PPP3R3C T cells with JNK inhibition (JNK‐IN‐8), which inhibit JNK pathway and phosphorylation of c‐Jun. We found that the expression of CD69, CD25 and CD40L were decreased in the JNK inhibition group compared to the sh‐PPP3R3C alone group following TCR/CD28 stimulation (Figure 3I), which was accompanied by decreased levels of IL‐2 and IFN‐γ protein levels (Figure S2G). These data establish that PPP2R3C played a modulatory role of TCR‐driven JNK signalling, with no discernible impact on the ERK or NF‐κB pathways, thereby fine‐tuning the activation thresholds of CD4^+^ T cells.

### 2.4 PPP2R3C functions as an inhibitor of abnormal activation of CD4^+^ T cells in SLE patients

CD4+ T cells from SLE patients display heightened activation upon TCR engagement. After isolating CD4+ T cells from PBMCs of 10 SLE patients and 10 HCs, we stimulated the purified cells with anti‑CD3/CD28 antibodies for 24 or 72 h. Activation markers (CD69, CD40L, CD25 and HLA‑DR) were significantly elevated in the SLE group relative to HCs (Figure S3A). Cytokine levels in 24‑h supernatants were measured using a flow cytometry‑based CBA kit, which indicated enhanced secretion of IL‑2, IL‑17, TNF‑α, IL‑4 and IFN‑γ from SLE patient cultures (Figure S3B). Moreover, as shown in Figure S4C, CD4^+^ T cells isolated from untreated SLE patients (*n* = 4) exhibited significantly elevated c‑Jun, P‑c‑Jun and P‑JNK upon anti‑CD3/CD28 stimulation compared to HCs (Figure S3C).

**FIGURE 4:**
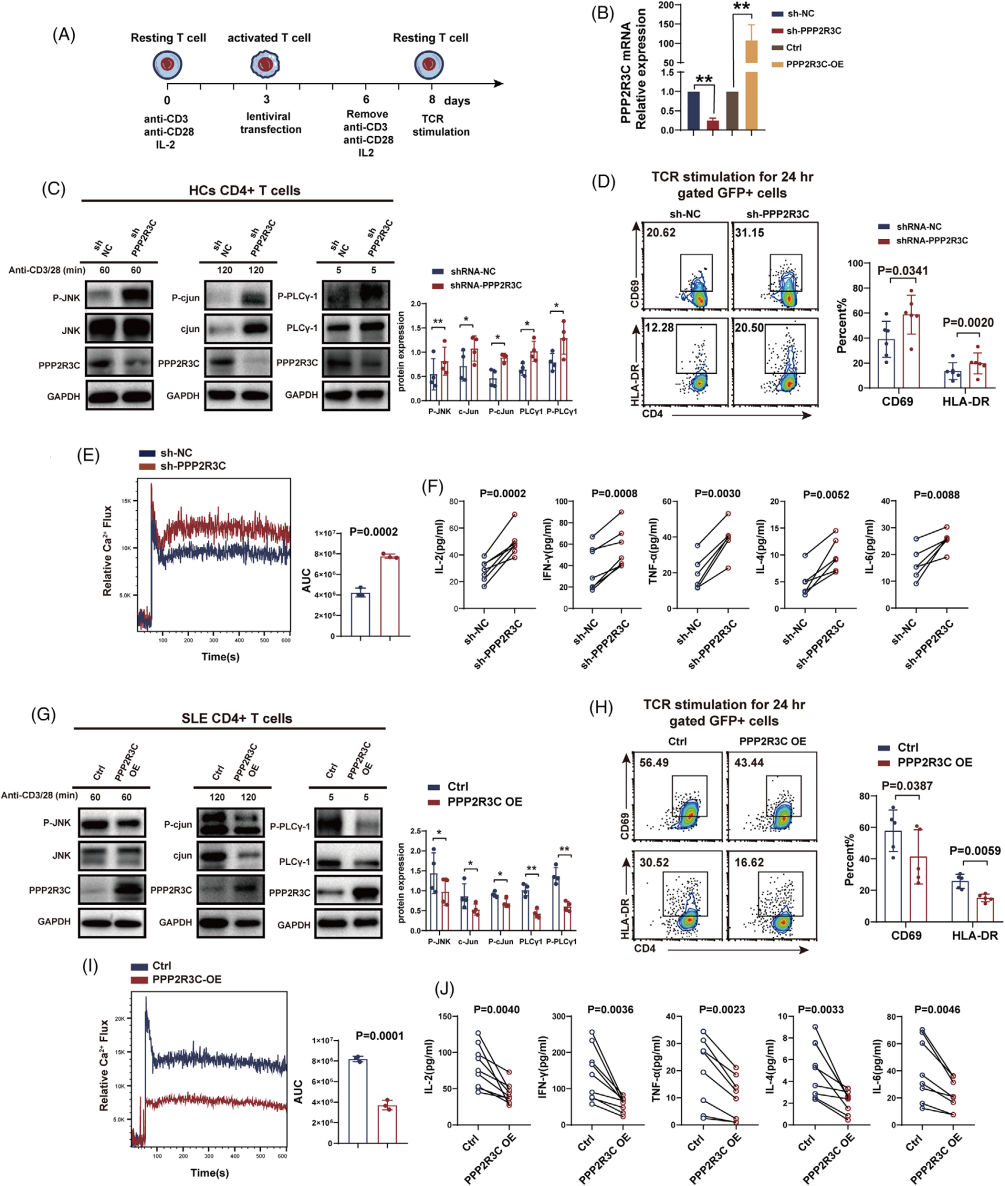
PPP2R3C functions as an inhibitor of abnormal activation of CD4^+^ T cells in systemic lupus erythematosus (SLE) patients. (A) Schematic of the lentiviral transduction protocol for primary human CD4^+^ T cells. (B) PPP2R3C knockdown or overexpression efficiency in healthy controls (HCs) and SLE CD4^+^T cells assessed by qRT‐PCR (*n* = 3). (C) Phosphorylated and total protein levels of PLCγ1, JNK and c‐Jun in sh‐PPP2R3C versus control HC CD4^+^ T cells at different time points after anti‐CD3 (5 µg/mL) and anti‐CD28 antibodies (2 µg/mL) stimulation (*n* = 4). (D) Analysis of CD69 and HLA‐DR surface expression on sh‐PPP2R3C versus control HC CD4^+^ T cells following 24‐h after anti‐CD3 (5 µg/mL) and anti‐CD28 antibodies (2 µg/mL) stimulation (*n* = 6). (E) Intracellular Ca^2^^+^ flux in sh‐PPP2R3C versus control HC CD4^+^ T cells following 24‐h after anti‐CD3 (5 µg/mL) and anti‐CD28 antibodies (2 µg/mL) stimulation (*n* = 3). (F) Secreted levels of IL‐2, IFN‐γ, TNF‐α and IL‐4 from sh‐PPP2R3C versus control HC CD4^+^ T cells after 24‐h after anti‐CD3 (5 µg/mL) and anti‐CD28 antibodies (2 µg/mL) stimulation (*n* = 6). (G) Phosphorylated and total protein levels of PLCγ1, JNK and c‐Jun in PPP2R3C‐OE versus control SLE CD4^+^ T cells at different time points after anti‐CD3 (5 µg/mL) and anti‐CD28 antibodies (2 µg/mL) stimulation (*n* = 4). (H) Analysis of CD69 and HLA‐DR surface expression on PPP2R3C‐OE versus control SLE CD4^+^ T cells following 24‐h after anti‐CD3 (5 µg/mL) and anti‐CD28 antibodies (2 µg/mL) stimulation (*n* = 5). (I) Intracellular Ca^2^^+^ flux in PPP2R3C‐OE and control SLE CD4^+^ T cells following 24‐h after anti‐CD3 (5 µg/mL) and anti‐CD28 antibodies (2 µg/mL) stimulation (*n* = 3). (J) Secreted levels of IL‐2, IFN‐γ, TNF‐α and IL‐4 from PPP2R3C‐OE versus control SLE CD4^+^ T cells after 24‐h after anti‐CD3 (5 µg/mL) and anti‐CD28 antibodies (2 µg/mL) stimulation (*n* = 8). Data are from a single experiment representative of at least two independent experiments. Data are presented as mean ± SD. *p* values were calculated by paired two‑tailed Student's *t*‑test (* indicates *p* < .05, ** indicates *p* < .01, *** indicates *p* < .001, ns, not significant).

To elucidate the role of PPP2R3C in this dysregulation, we engineered PPP2R3C knockdown (sh‐PPP2R3C) in HCs CD4^+^ T cells and overexpression (PPP2R3C‐OE) in SLE CD4^+^ T cells using lentiviral vectors. Given the low transfection efficiency in quiescent primary T cells, we primed the cells with anti‐CD3/CD28 and IL‐2 for 72 h prior to transduction, followed by viral delivery and a 48‐h rest period to restore quiescence (Figure 4A). We confirmed successful modulation of PPP2R3C in CD4^+^ T cells (Figure 4B).

In HC CD4^+^ T cells, following anti‑CD3/CD28 stimulation, PPP2R3C knockdown significantly increased both total and phosphorylated PLCγ1 (Tyr783) and enhanced JNK/c‐Jun phosphorylation (Figure 4C). Consistent with a heightened activation state, sh‑PPP2R3C HC CD4^+^ T cells also exhibited increased expression of activation markers CD69 and HLA‑DR (Figure 4D), amplified calcium (Ca^2^^+^) influx (Figure 4E) and elevated secretion of IL‑2, IFN‑γ, TNF‑α and IL‑4 (Figure 4F).

In contrast, PPP2R3C overexpression in SLE CD4^+^ T cells reversed the hyperactivation phenotype, reducing total and phosphorylated PLCγ1 levels and suppressing JNK/c‐Jun phosphorylation (Figure 4G). This was accompanied by decreased expression of activation markers (Figure 4H), attenuated calcium influx (Figure 4I) and reduced cytokine production (Figure 4J).

To further assess whether PPP2R3C modulates additional downstream signalling pathways in primary CD4^+^ T cells, we examined p65 and ERK phosphorylation upon TCR stimulation. Consistent with our observations in Jurkat cells, we found no reproducible alterations in p65 or ERK phosphorylation upon PPP2R3C modulation (Figure S4D).

These findings identify PPP2R3C as a critical regulator of T cell hyperactivation in SLE, acting primarily through the PLCγ‑1–JNK/c‑Jun axis without significantly affecting the NF‑κB or ERK pathways.

### 2.5 Tissue and cell‐type‐specific dysregulation of PPP2R3C in PIL mice

Pristane instigates disease by fostering an inflammatory environment through macrophage activation and the production of reactive intermediates, as well as generating autoantigens via apoptosis. Collectively, these processes promote aberrant T and B cell activation, ultimately leading to organ damage.^18^, ^19^ To delineate the expression of PPP2R3C in PIL mice, a murine model of systemic autoimmunity was established through intraperitoneal administration of pristane in BALB/c mice. The PIL mice exhibited hallmark pathological features, including peritoneal inflammation, production of antinuclear antibodies (ANAs) and glomerulonephritis.^20^ After 24 weeks pristane treated, immunophenotyping revealed a significant disruption of immune homeostasis, characterised by elevated serum levels of anti‐dsDNA antibodies (Figure S4A), total IgG titres (Figure S4B) and ANA titres (Figure S4D). Consistent with renal pathology, PIL mice demonstrated pronounced proteinuria (Figure S4C). Pathologically, PIL mice exhibited glomerular deposition of IgG and complement C3 (Figure S4E). Haematoxylin and eosin (H&E) staining further revealed moderate mesangial hyperplasia in glomeruli, tubular dilation and vacuolar degeneration with occasional protein casts, as well as focal interstitial inflammatory cell infiltration, indicating more severe renal injury compared to wild‐type (WT) mice (Figure S4F).

**FIGURE 5:**
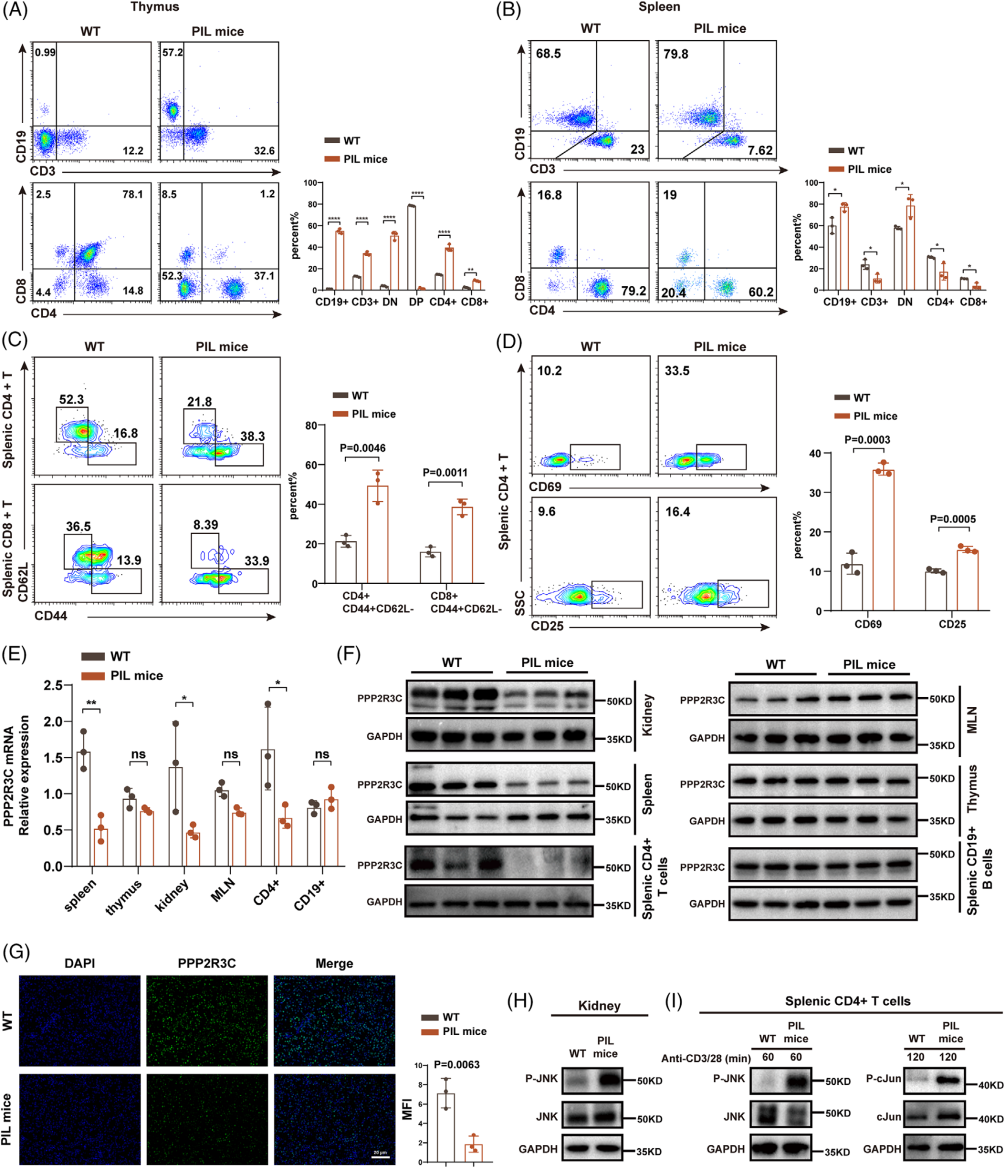
Tissue and cell‐type‐specific dysregulation of PPP2R3C in pristane‐induced lupus (PIL) mice. (A) Flow cytometric analysis of thymic immune cell populations in wild‐type (WT) and PIL mice, showing percents of CD19^+^ B cells, CD3^+^ T cells, DN T cells, DP T cells, CD4^+^ T cells and CD8^+^ T cells (*n* = 3). (B) Splenic immune cell subsets in WT and PIL mice, quantifying CD19^+^ B cells, DN T cells, CD3^+^ T cells, CD4^+^ T cells and CD8^+^ T cells (*n* = 3). (C) Proportions of activated T cell subsets (CD4^+^CD44^+^CD62L^−^ and CD8^+^CD44^+^CD62L^−^) in the spleen of WT and PIL mice (*n* = 3). (D) Surface expression of CD69 and CD25 on splenic CD4^+^ T cells from WT and PIL mice (*n* = 3). (E) Relative PPP2R3C mRNA levels in splenic CD4^+^ T cells, total spleen, kidney, mesenteric lymph nodes (MLNs), thymus, and CD19^+^ B cells from WT and PIL mice (*n* = 3). (F) PPP2R3C protein expression in the spleen, splenic CD4^+^ T cells, kidney, MLN, thymus and CD19^+^ B cells from WT and PIL mice (*n* = 3). (G) Immunofluorescence analysis of PPP2R3C protein expression (green) in kidney sections from WT and PIL mice (*n* = 3). (H) Western‐blot analysis of P‐JNK expression in kidney from WT and PIL mice (*n* = 3). (I) Western blot analysis of JNK/c‐Jun pathway activation in splenic CD4+ T cells from WT and PIL mice stimulated with anti‐CD3 (5 µg/mL) and anti‐CD28 (2 µg/mL) for 1 or 2 h (*n* = 3). Data from a single independent experiment are presented as mean ± SD. *p* values were calculated using unpaired two‐tailed Student's *t*‐test (* indicates *p* < .05, ** indicates *p* < .01, *** indicates *p* < .001, ns, not significant).

The autoimmune phenotype detected in PIL mice led us to thoroughly evaluate their immune system status relative to WT mice. We therefore analysed multiple immune cell subtypes, CD3^+^ T cells, CD19^+^ B cells, DP CD4^+^CD8^+^ cells, double‐negative (DN) CD4^−^CD8^−^ cells, CD4^+^ T cells and CD8^+^ T cells, in both the thymus and spleen. In the thymus of PIL mice, we observed a notable rise in the percentages of CD19^+^ B cells, CD3^+^ T cells, DN cells and CD4^+^ T cells, along with a significant drop in DP cell proportions. The autoimmune phenotype observed in PIL mice prompted an investigation into the status of their immune system in comparison to WT. Then, we comprehensively analysed various immune cell subtypes, including CD3^+^T cells, CD19^+^ B cells, CD4^+^CD8^+^ DP cells, CD4^−^CD8^−^ DN cells, CD4^+^ T cells and CD8^+^ T cells, within the thymus and spleen. We observed a significant increase in the proportions of CD19^+^ B cells, CD3^+^ T cells, DN cells and CD4^+^ T cells within the thymus of PIL mice, accompanied by a marked reduction in DP cells (Figure 5A). Similarly, the analysis of splenic immune cell populations demonstrated an elevated percentage of CD19^+^ B cells and DN cells, while the proportions of CD3^+^ T cells, CD4^+^ T cells and CD8^+^ T cells were notably decreased (Figure 5B). Furthermore, enhanced T cell activation was noted in both CD4^+^ and CD8^+^ T cell compartments, as evidenced by a substantial rise in the proportions of CD44^+^CD62L^−^CD4^+^ and CD44^+^CD62L^−^CD8^+^ T cell populations, and elevated expression of the activation markers CD69 and CD25 on splenic CD4^+^ T cells within the spleen of PIL mice (Figure 5C,D). Similar to observations in SLE patients, PPP2R3C mRNA in the splenic CD4^+^ T cells of PIL mice were significantly downregulated. Additionally, we noted a decrease in PPP2R3C mRNA levels in both the spleen and kidney of PIL mice, while no significant alterations were detected in the mesenteric lymph nodes (MLNs), thymus, or CD19^+^ B cells (Figure 5E). Consistent with these findings, PPP2R3C protein expression was also reduced in the spleen, splenic CD4^+^ T cells and kidney of PIL mice, whereas no alterations were observed in other cell subsets (Figure 5F). Strikingly, PPP2R3C was found to reside primarily within the nuclear compartment of renal cells, a localisation readily detectable in both glomeruli and tubules. Immunofluorescence co‑localisation analysis further revealed that PPP2R3C is expressed in podocytes (Nephrin^+^), endothelial cells (CD31^+^) and mesangial cells (PDGFR‑β^+^). Notably, in PIL mice, PPP2R3C expression was significantly downregulated in all three cell types compared to WT controls (Figures 5G and S4G). Consistent with the characteristic features of PIL nephritis, PIL mice also exhibited marked mesangial cell proliferation. Moreover, renal p‑JNK levels were markedly increased in PIL mice (Figure 5H). Collectively, these findings demonstrate that PPP2R3C is broadly downregulated in multiple kidney resident cell populations in lupus‑like kidney injury, accompanied by increased p‑JNK activation and mesangial proliferation.

We further examined JNK/c‑Jun pathway activation in CD4^+^ T cells from WT and PIL mice. Following anti‑CD3/CD28 stimulation, PIL mouse CD4^+^ T cells exhibited significantly increased phosphorylation of JNK and c‑Jun, as well as elevated total c‑Jun levels, compared to WT controls (Figure 5I).

Together, these results reveal a tissue‐ and cell‐type‑specific dysregulation of PPP2R3C in PIL mice, characterised by its broad downregulation in both lymphoid tissues and kidney resident cells, accompanied by hyperactivation of the JNK/c‑Jun pathway in CD4^+^ T cells and increased renal p‑JNK levels. This pattern suggests that PPP2R3C deficiency may contribute to lupus pathogenesis through both immune and renal compartment‑intrinsic mechanisms.

### 2.6 T cell‐targeted PPP2R3C reconstitution via Ark313 confers long‐term protection in SLE

Given the observed downregulation of PPP2R3C in CD4^+^ T cells from SLE patients and PIL mice. We hypothesised that restoring PPP2R3C expression in T cells could mitigate lupus‐associated immunopathology. To test this, we employed the engineered adeno‐associated virus (AAV) variant Ark313, a platform validated for in vivo T cell gene editing and large DNA template delivery, including integration into naive CD4^+^ and CD8^+^ T cells.^21^ Mice received dual‐phase intravenous administrations of Ark313‐PPP2R3C overexpression (Ark313‐PPP2R3C) or Ark313‐ctrl (non‐targeting control), consisting of a priming dose administered 7 days prior to the pristane challenge and a booster dose given 10 weeks post‐induction. Disease progression was monitored at 24 and 48 weeks (Figure S5A). After 24 weeks of Ark313‐PPPP2R3C treatment, the expression of PPP2R3C was significantly elevated in the spleen and in splenic CD4^+^ and CD8^+^ T cells, while no such elevation was observed in renal tissue, thymus, or MLN (Figure S5B).

**FIGURE 6:**
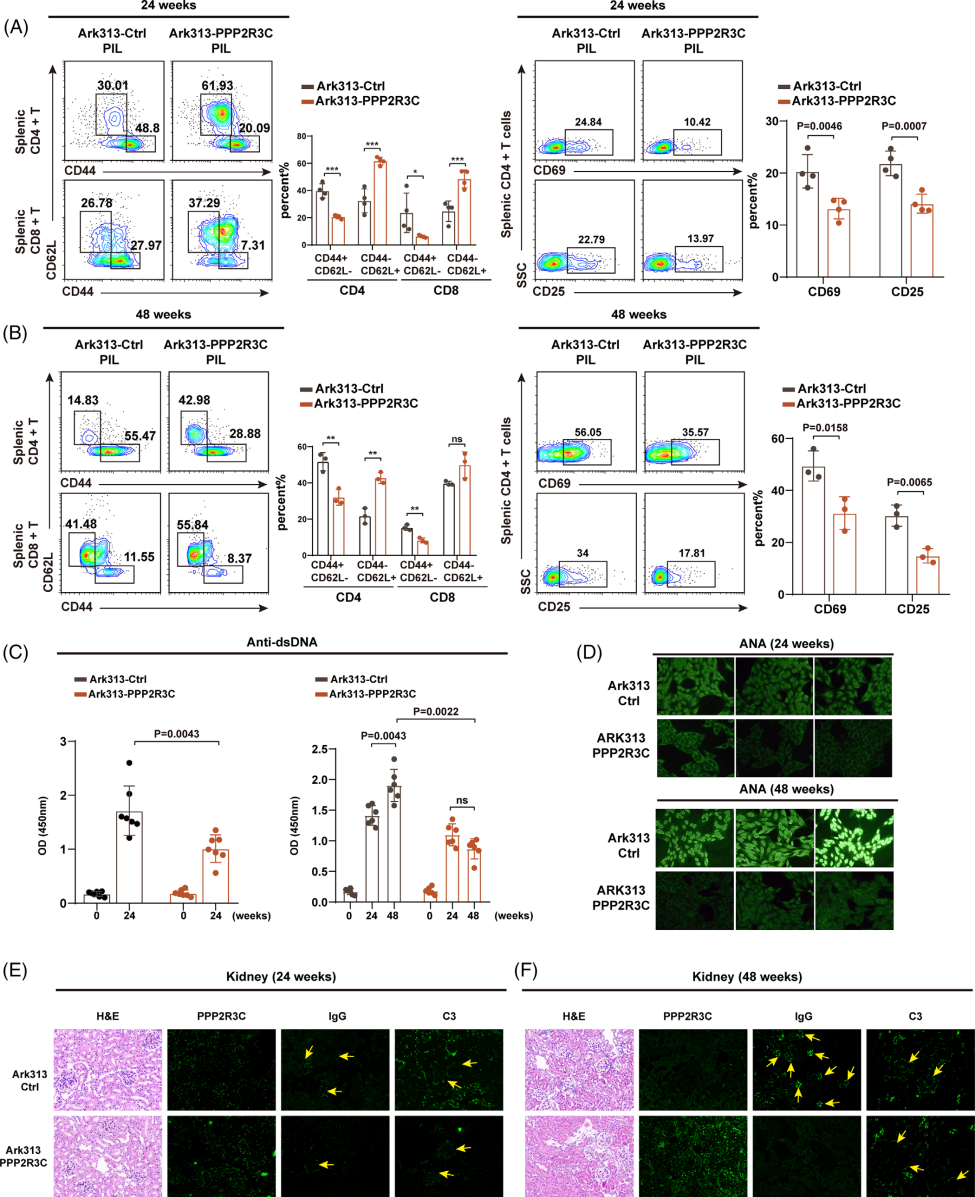
T cell‐targeted PPP2R3C reconstitution via Ark313 confers long‐term protection in systemic lupus erythematosus (SLE). (A, B) Flow cytometric analysis of activated splenic T cell subsets (CD44^+^CD62L^−^ CD4^+^ and CD44^+^CD62L^−^CD8^+^T cells) and activation markers (CD69 and CD25) on CD4^+^T cells in Ark313‐PPP2R3C and Ark313‐ctrl mice at (A) 24 weeks (*n* = 4) and (B) 48 weeks post‐pristane induction (*n* = 3). (C) Serum anti‐dsDNA antibody titres in Ark313‐PPP2R3C and Ark313‐ctrl mice at 24 (*n* = 7) and 48 weeks (*n* = 6). (D) Representative images of antinuclear antibody (ANA) staining patterns in serum from Ark313‐PPP2R3C and Ark313‐ctrl mice at 24 and 48 weeks. (E, F) Representative kidney haematoxylin and eosin (H&E) staining, PPP2R3C, C3 and total IgG staining from Ark313‐PPP2R3C and Ark313‐ctrl mice at (E) 24 weeks (*n* = 4) and (F) 48 weeks (*n* = 3). Data from a single independent experiment are presented as mean ± SD. *p* values were calculated using unpaired two‐tailed Student's *t*‐test (* indicates *p* < .05, ** indicates *p* < .01, *** indicates *p* < .001, ns, not significant).

To determine whether this restricted overexpression altered the immune landscape, we next analysed major lymphocyte subsets. At both 24 and 48 weeks, no significant differences were detected in thymic immune cell subsets between Ark313‐PPP2R3C and Ark313‐ctrl mice (Figure S5C). In the spleen, a modest yet consistent reduction in CD19^+^ B cells and DN T cells were noted at both time points, alongside a partial restoration of CD3^+^, CD4^+^ and CD8^+^ T cell populations; however, these changes did not reach statistical significance (Figure S5D). In contrast, by 24 weeks, Ark313‐PPP2R3C mice exhibited significantly reduced levels of activated T cell subsets (CD44^+^CD62L^−^CD4^+^ and CD44^+^CD62L^−^CD8^+^ T cells) as well as decreased surface expression of CD69 and CD25 on splenic CD4+ T cells (Figure 6A). Notably, at the 48‐week time point, Ark313‐PPP2R3C treatment maintained suppression of T cell activation, whereas disease progression in control mice resulted in enhanced T cell activation (Figure 6B). Systemic autoimmunity was evaluated through serum levels of anti‐dsDNA and ANAs. At both 24 and 48 weeks, all of Ark313‐PPP2R3C mice exhibited reduced titres of anti‐dsDNA compared to controls (Figure 6C). Although the anti‐dsDNA levels in the Ark313‐PPP2R3C treatment group showed a declining trend between 24 and 48 weeks, this was not statistically significant, while Ark313‐ctrl mice demonstrated a marked increase, consistent with disease progression. Interestingly, at 24 weeks, ANA staining revealed a nuclear pattern in Ark313‐Ctrl mice, whereas a cytoplasmic pattern was observed in Ark313‐PPP2R3C mice. By 48 weeks, Ark313‐PPP2R3C mice displayed cytoplasmic positivity with weak nuclear staining, in contrast to controls, which exhibited intensified nuclear fluorescence (Figure 6D). Renal pathology exhibited progressive improvement overtime.

Although no significant intergroup differences in proteinuria, H&E score or renal IgG/C3 deposition were observed at 24 weeks (Figures 6E and S5E),

Ark313‑PPP2R3C‑treated mice exhibited a significant reduction in both parameters compared to control groups at the 48‑week assessment (Figures 6F and S5F). This recovery was associated with suppressed p‑JNK activation in both the kidney and CD4^+^ T cells at 48 weeks (Figure S5G,H), indicating that targeting T cell activation through PPP2R3C may represent a promising therapeutic strategy for SLE. Although the intervention did not ameliorate renal injury at the early stage (24 weeks), it significantly reduced kidney damage by the late disease phase (48 weeks). These results demonstrate that PPP2R3C overexpression provides sustained suppression of autoimmune progression and offers end‑organ protection by modulating T cell activation and autoantibody responses.

### 2.7 AAV9‐PPP2R3C delivery rescued multi‐organ lupus pathology in PIL mice

**FIGURE 7:**
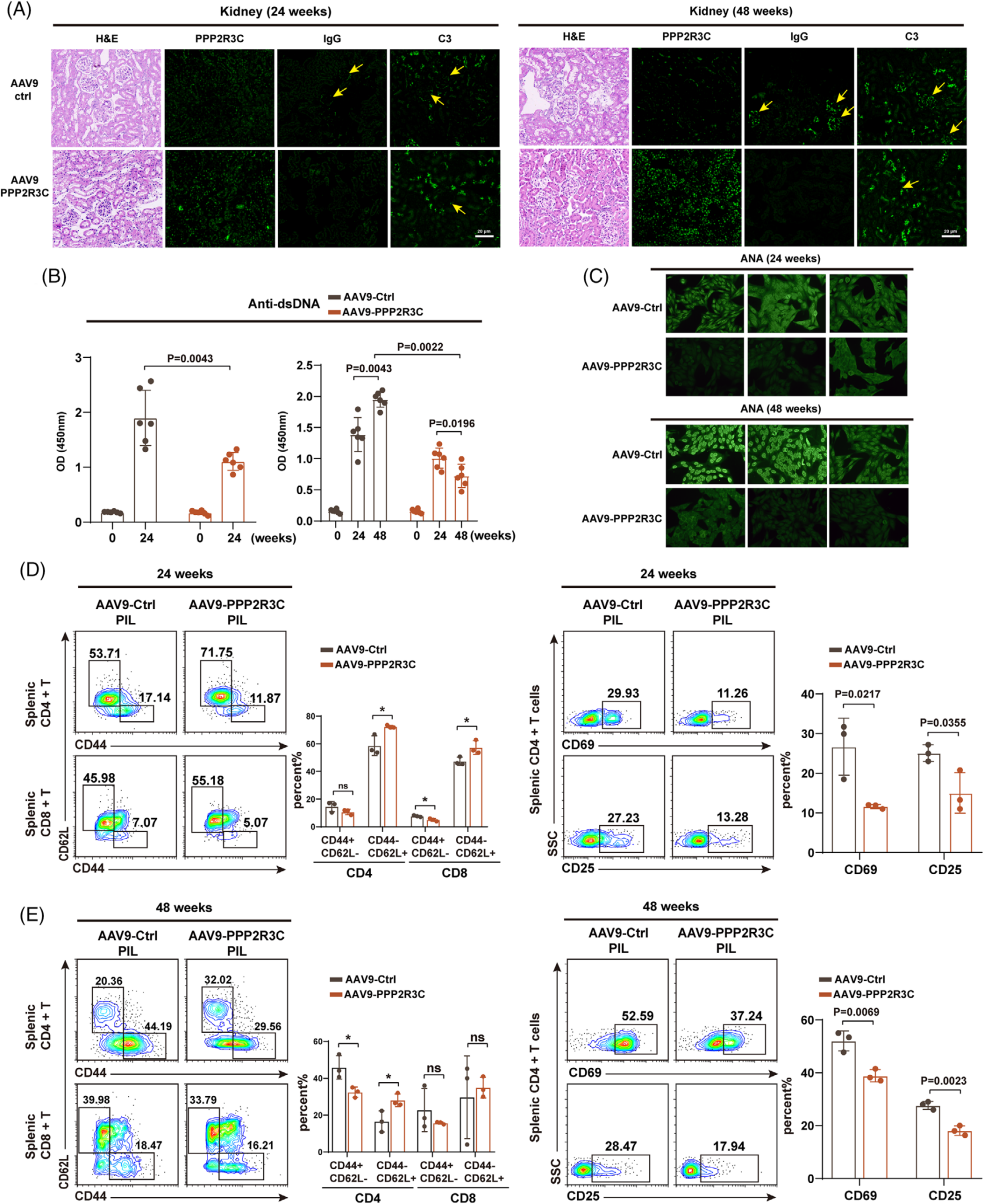
Systemic PPP2R3C delivery rescued multi‐organ lupus pathology in pristane‐induced lupus (PIL) mice. (A) Representative haematoxylin and eosin (H&E) staining and immunofluorescence staining for PPP2R3C, C3 and total IgG in kidney sections from AAV9‐ctrl and AAV9‐PPP2R3C‐treated PIL mice at 24 and 48 weeks (*n* = 3). (B) Serum anti‐dsDNA antibody levels in AAV9‐ctrl and AAV9‐PPP2R3C‐treated PIL mice at 24 and 48 weeks post‐pristane induction (*n* = 3). (C) Serum antinuclear antibody (ANA) titres in AAV9‐ctrl and AAV9‐PPP2R3C‐treated PIL mice at 24 weeks and 48 weeks (*n* = 6). (D, E) Flow cytometric analysis of activated splenic T cell subsets (CD44^+^CD62L^−^CD4^+^ and CD44^+^CD62L^−^CD8^+^ T cells) and activation markers (CD69 and CD25) on CD4^+^ T cells in AAV9‐ctrl and AAV9‐PPP2R3C‐treated mice at (D) 24 weeks and (E) 48 weeks (*n* = 3). Data from a single independent experiment are presented as mean ± SD. *p* values were calculated using unpaired two‐tailed Student's *t*‐test (* indicates *p* < .05, ** indicates *p* < .01, *** indicates *p* < .001, ns, not significant).

Our initial findings in PIL mice indicated a downregulation of PPP2R3C expression in both the spleen and kidney. Based on these observations, we hypothesised that the simultaneous overexpression of PPP2R3C in these two organs could enhance therapeutic efficacy. To evaluate this hypothesis, we administered AAV9‐PPP2R3C overexpression (AAV9‐PPP2R3C) and AAV9‐ctrl systemically via tail vein injection, following the same regimen used for ARK313 (Figure S6A). After 24 weeks of pristane exposure, we confirmed sustained overexpression of PPP2R3C in the spleen and kidney, while no significant expression was detected in the thymus or MLN (Figure S6B). We next evaluated the effect of AAV9‑PPP2R3C on SLE progression at 24 and 48 weeks. Renal immunofluorescence analysis revealed that AAV9‑PPP2R3C treatment induced sustained PPP2R3C reconstitution, accompanied by reduced glomerular deposition of IgG/C3 (Figure 7A), and decreased proteinuria (Figure S6C). Consistently, H&E staining demonstrated ameliorated renal injury at both 24 and 48 weeks, including reduced mesangial hyperplasia, tubular damage and interstitial inflammation (Figure 7A). Quantitative histologic scoring and immunofluorescence intensity (Figure S6D) confirmed significant renal protection. Notably, the therapeutic effect became more pronounced by 48 weeks, indicating a time‑dependent enhancement of renal preservation. Serum levels of anti‐dsDNA antibodies (Figure 7B) and ANA titres (Figure 7C) were significantly lower in AAV9‐PPP2R3C treated mice than in controls at both time points. Notably, the therapeutic effect became more pronounced by 48 weeks, indicating a time‐dependent enhancement of renal preservation. Serum levels of anti‐dsDNA antibodies (Figure 7B) and ANA titres (Figure 7C) were significantly lower in AAV9‐PPP2R3C‐treated mice than in controls at both time points. Longitudinal tracking of anti‐dsDNA levels revealed a pronounced decline between 24 and 48 weeks in the treatment group, contrasting with a progressive increase in controls. Similarly, ANA staining at 48 weeks showed intense fluorescence in controls, whereas the AAV9‐PPP2R3C group maintained only weak positivity. These findings underscore the sustained immunomodulatory effects of the therapy.

To determine whether AAV9‐PPP2R3C promotes immune restoration, immunophenotyping was conducted at 24 and 48 weeks. In contrast to ARK313‐treated mice, thymic analysis of AAV9‐PPP2R3C PIL mice showed a significant reduction in CD19^+^ B cells, CD3^+^ T cells, CD4^+^ T cells and DN T cells, along with a recovery of DP thymocytes at both time points (Figure S6E). In the spleen, a marked decrease in DN T cells and partial restoration of CD3^+^ and CD4^+^ T cells were observed. CD19^+^ B cells were significantly reduced, and CD8^+^ T cells increased notably by 48 weeks (Figure S6F). Furthermore, the activated CD8^+^ T cell subset (CD44^+^CD62L^−^CD8^+^) and the activation markers CD69 and CD25 on splenic CD4^+^ T cells were significantly suppressed in AAV9‑PPP2R3C‑treated mice at 24 weeks. In contrast, the activated CD4^+^ T cell subset (CD44^+^CD62L^−^CD4^+^) showed a decreasing trend that did not reach statistical significance. This is likely attributable to the limited sample size, which may have resulted in insufficient statistical power to detect a true difference (Figure 7D). Significant downregulation of CD44^+^CD62L^−^CD4^+^ T cells and CD69/CD25 activation markers on CD4^+^ T cells were maintained at 48‐week. Although the difference in CD44^+^CD62L^−^CD8^+^ T cells was no longer statistically significant (Figure 7E).

To directly assess the impact of AAV9‑PPP2R3C on JNK/c‑Jun signalling, we isolated splenic CD4^+^T cells at 48 weeks and stimulated them with anti‐CD3/CD28. Compared to control‑treated PIL mice, CD4^+^T cells from AAV9‑PPP2R3C mice exhibited significantly reduced phosphorylation of JNK and c‑Jun, as well as lower total c‑Jun levels (Figure S6G). Consistently, in the kidney, AAV9‑PPP2R3C treatment also markedly reduced p‑JNK levels at 48 weeks (Figure S6H). These results demonstrate that AAV9‑PPP2R3C treatment not only restores PPP2R3C expression but also dampens the hyperactivated JNK/c‑Jun pathway in both CD4^+^T cells and renal tissue, aligning with the observed reduction in T cell activation, renal injury and autoantibody production.

## 3 DISCUSSION

PP2A is a critical serine/threonine phosphatase complex that regulates diverse cellular processes through the dephosphorylation of numerous substrates.^22^ Among its regulatory subunits, PPP2R3C (also known as G5PR) is highly expressed in thymocytes, where it contributes to apoptosis via JNK‐mediated caspase‐3 activation.^15^ Our study extends this paradigm to peripheral T cells, identifying PPP2R3C as a selective modulator of TCR‐induced JNK signalling and a pivotal regulator of immune tolerance. We demonstrate that PPP2R3C expression is selectively downregulated in CD4^+^ T cells from SLE patients, correlating with disease severity, and that this deficiency drives pathogenic T cell hyperactivation and tissue injury.

The TCR signalling cascade is a foundation of adaptive immunity, governing T cell activation, differentiation and self‐tolerance.^23^ Dysregulation of this pathway underlies both immunodeficiency and autoimmune pathologies.^24^ As a key negative regulator, PP2A restrains T cell activation through specific regulatory subunits. For instance, Zhou et al.^9^ demonstrated that suppression of PPP2R2D enhances the function of effector CD4^+^/CD8^+^ T cells and tumour‐infiltrating lymphocytes, thereby augmenting antitumour immunity. Mechanistically, PP2A associates with its regulatory subunit PPP2R1A to dephosphorylate Carma1 at Ser645, limiting the Carma1–Bcl10–Malt1 (CBM) complex formation and subsequent NF‐κB activation, which ultimately constrains TCR‐induced production of IL‐2 and IFN‐γ.^25^ Similarly, B56γ (PPP2R5C) silencing increases IKK/IκBα phosphorylation and NF‐κB activity, elevating IL‐2 expression and T cell proliferation.^8^ Together, these studies establish PP2A as a pivotal suppressor of TCR‐driven NF‐κB signalling through subunit‐specific mechanisms. In contrast to these NF‐κB directed mechanisms, our findings indicate that PPP2R3C operates through a distinct signalling axis in SLE. The JNK pathway, a key branch of the MAPK signalling network, controls cellular growth, survival and apoptosis, and is essential for regulating T and B cell immune responses.^26^, ^27^, ^28^ While the activation of JNK pathway through phosphorylation upon TCR stimulation is well‐documented,^29^, ^30^ the mechanisms involved in the termination and fine‐tuning of TCR signalling through dephosphorylation are not fully understood. Given the limited understanding of the negative regulation of JNK signalling, its critical role in maintaining immune homeostasis becomes particularly evident. The pathway influences various phases of the immune response, beginning with T cell selection in the thymus and extending to the development of self‐tolerance in mature cells beyond the thymus.^30^ Furthermore, the JNK pathway is linked to several abnormalities in a range of autoimmune diseases, such as inflammatory bowel disease, and SLE.^31^, ^32^ In SLE patient, heightened JNK activity has been associated with increased disease activity and long‐term damage to organs.^33^ In addition, targeting JNK with D‑JNKi, a peptide inhibitor, substantially reduced inflammation and joint destruction in mice with antigen‑induced arthritis.^34^ The use of JNK inhibitors has been shown to significantly mitigate lupus‐like symptoms by inhibiting the pyroptosis of renal tubular epithelial cells.^35^

Our findings position PPP2R3C as a critical negative regulator that constrains TCR signalling at a proximal level. Specifically, PPP2R3C deficiency in SLE CD4^+^ T cells leads to increased total and phosphorylated PLCγ1 (Tyr783), whereas PPP2R3C overexpression reduces both. We acknowledge that the effect on total PLCγ1 levels is unexpected for a phosphatase regulatory subunit. Our RNA‑seq data did not reveal significant changes in PLCγ1 mRNA levels, suggesting that the regulation occurs at a post‑transcriptional level, possibly via protein stability or translation efficiency. The detailed mechanism remains to be elucidated and will be pursued in future studies. This regulation of PLCγ1 directly impacts calcium flux and downstream JNK activation. We further demonstrate that the enhanced JNK phosphorylation upon PPP2R3C loss is dependent on calcium flux, as chelation of intracellular calcium with BAPTA‐AM abrogates this effect. Thus, PPP2R3C acts upstream of the PLCγ1–calcium–JNK axis, selectively dampening JNK/c‑Jun phosphorylation without altering ERK or NF‑κB pathway activity. Through this targeted modulation, PPP2R3C enforces T cell quiescence and prevents sustained activation, thereby maintaining immune homeostasis.

Importantly, JNK/c‐Jun is not the only pathway downstream of PPP2R3C. In Jurkat T cells, we also observed that PPP2R3C knockdown enhances AKT/mTOR phosphorylation (data not shown), consistent with its upstream regulation of PLCγ1, which can activate the PI3K–AKT axis via DAG‐PKCθ‐CBM signalosome.^36^ However, this finding was not consistently validated in primary CD4^+^ T cells from SLE patients and HCs, highlighting potential differences between immortalised cell lines and primary human T cells, as well as the inherent variability of patient samples. Therefore, while AKT/mTOR may represent an additional pathway modulated by PPP2R3C in certain cellular contexts, its relevance in primary human T cells and in lupus pathogenesis remains to be determined. The present study focuses on the JNK/c‐Jun axis as a major and consistently validated effector in both Jurkat and primary T cells, but we acknowledge that PPP2R3C may exert broader signalling effects that warrant future investigation.

Nevertheless, we acknowledge that our current study does not provide direct biochemical evidence, such as co‐immunoprecipitation or in vitro phosphatase assays, demonstrating that PPP2R3C‐containing PP2A holoenzymes physically interact with or directly dephosphorylate JNK or c‑Jun. The observed regulation could be mediated through indirect mechanisms involving upstream regulators like MAP3K1, which has been shown to be antagonised by PPP2R3C,^37^ or through the interaction of PPP2R3C with PLCγ1 identified in our study. Detailed mechanistic dissection, including direct biochemical validation and structure–function analyses, will be pursued in future studies.

In vivo studies confirmed a concomitant, tissue‐specific downregulation of PPP2R3C in both CD4^+^ T cells and kidney of PIL mice, linking its deficiency to dual pathology: systemic immune hyperactivation and glomerular injury. To exploit this precision, we engineered T cell‐targeted PPP2R3C overexpression using the engineered AAV variant Ark313,^21^, ^38^, ^39^ which normalised splenic T cell hyperactivation response at both 24 weeks and 48 weeks. This intervention diminished activation markers such as CD69 and CD25 in splenic CD4^+^ T cells, and reduced serum autoantibodies (ANA and anti‐dsDNA). The renal protection conferred by Ark313‐PPP2R3C underscores the pivotal role of systemic immune modulation in mitigating end‐organ damage, albeit with a delayed onset. The initial absence of therapeutic efficacy on renal pathology, including proteinuria and immune complex deposition, likely stems from the combined effects of limited renal transduction due to the splenic T‐cell tropism of Ark313. In the multifaceted pathogenesis of LN, infiltrating immune cells contribute substantially to the disease^40^, ^41^, ^42^; however, resident renal cells, including tubular epithelial cells, mesangial cells and podocytes, also actively participate in disease progression.^43^, ^44^, ^45^ The delivery limitations of Ark313's tropism for splenic T cells hindered renal transduction, leaving tissue‐resident inflammatory pathways unaffected. Accordingly, no renal transgene upregulation or therapeutic advantage was observed at 24 weeks, a time when baseline injury remained modest. The protective effect of Ark313‐PPP2R3C emerged by 48 weeks. This delayed therapeutic divergence coincided with sustained systemic immunomodulation. These findings support an indirect mechanism of nephroprotection via attenuation of the systemic autoimmune response. Thus, while control animals developed progressive nephritis, Ark313‐PPP2R3C treated mice, buffered from escalating immunopathology, displayed significantly preserved renal structure and function.

In contrast, AAV9‐mediated systemic delivery of PPP2R3C achieved a broader tissue distribution, including the kidney and spleen.^46^ This approach produced earlier and more comprehensive therapeutic effects, ameliorating multi‐organ pathology and reducing autoimmune markers as early as 24 weeks, with effects sustained at 48 weeks. Immunofluorescence co‐localisation revealed that PPP2R3C is expressed in podocytes, endothelial cells and mesangial cells in mice, and its expression is significantly reduced in PIL kidneys. The superior efficacy of AAV9‐PPP2R3C raises the possibility that direct restoration of PPP2R3C in kidney resident cells may contribute additional renoprotective effects, independent of its immunomodulatory functions. This is consistent with the observed reduction in renal p‐JNK levels upon PPP2R3C restoration. However, we acknowledge that causal evidence for a cell‐intrinsic renal protective role is lacking; future studies using cell‐type‐specific conditional gain‐ or loss‐of‐function approaches are required to definitively dissect the contributions of PPP2R3C in different renal compartments.

Prior studies have established that PPP2R3C deficiency triggers apoptosis via JNK hyperactivation.^15^ Consistently, we found that PPP2R3C knockdown increased TUNEL^+^Jurkat T cells, while overexpression reduced apoptosis (data not shown). In PIL mice, splenic CD4^+^T cell percentages were reduced, and PPP2R3C restoration significantly increased these percentages while concurrently reducing p‐JNK levels. Given the well‐established role of sustained JNK activation in activation‐induced cell death (AICD),^47^ the most parsimonious interpretation is that the increased T cell numbers reflect reduced apoptosis. Future studies using T‐cell‐specific conditional knockout mice will be required to directly test the contribution of PPP2R3C to T cell apoptosis in vivo.

Beyond its effects on peripheral T cell survival, PPP2R3C restoration also modulated central immune compartments. In contrast to Ark313, AAV9‐mediated PPP2R3C overexpression elicited a distinct immunomodulatory effect within the thymus, characterised by the normalisation of CD19^+^ B cell, DN T cell and DP T cell populations, further underscores the systemic immunomodulatory capacity of PPP2R3C. Although systemic overexpression of PPP2R3C may lead to improved therapeutic outcomes, the broader tissue tropism of AAV9 raises considerations regarding potential off‐target effects or immunotoxicity. Longitudinal studies are warranted to evaluate the chronic safety and durability of this approach, balancing targeted immunomodulation with global immune compromise.

Our data also reveal functional divergence among PP2A regulatory subunits in immune regulation. While PPP2R2A has been shown to promote systemic autoimmunity through NAD^+^‐dependent metabolic reprogramming,^12^, ^13^ PPP2R3C exerts the opposite effect, suppressing autoimmunity by selectively attenuating TCR‐PLCγ1‐JNK signalling. This specialised function of individual subunits enables the PP2A complex to precisely target distinct molecular pathways, a strategy that is essential for maintaining immune equilibrium. Understanding these discrete roles opens new avenues for precision‐targeted immunotherapy that restores immune balance without broad immunosuppression.

In summary, our study identifies PPP2R3C as a critical phosphatase subunit that safeguards immune tolerance by selectively restraining JNK/c‐Jun signalling downstream of the TCR, in part through calcium‐dependent mechanisms. While JNK/c‐Jun represents a major and consistently validated effector, it may not be the only pathway modulated by PPP2R3C, as additional signalling nodes such as AKT/mTOR showed regulation in Jurkat cells. Loss of PPP2R3C expression in SLE disrupts this regulatory axis, driving T cell hyperactivation, and autoantibody production. Genetic restoration of PPP2R3C expression reverses these defects, mitigates systemic autoimmunity and confers renal protection. These findings establish PPP2R3C as a key molecular checkpoint in the PP2A network and a promising therapeutic target for restoring immune homeostasis in lupus.

### 3.1 Limitations of the study

While our findings establish PPP2R3C as a critical negative regulator of T cell hyperactivity and a promising therapeutic target in lupus, this study has several limitations.

We acknowledge that the number of patients with LN in our cohort is relatively small (*n* = 14), which may limit the statistical power of our subgroup analyses. We have obtained preliminary renal immunofluorescence data from three LN patients and three disease controls, indicating reduced PPP2R3C expression in lupus kidneys. However, larger cohorts are needed to enable stratification by disease class and activity. In future work, we plan to expand the sample size and to analyse PPP2R3C expression according to LN class, which will help establish its clinical and pathological relevance.

Despite the robust functional evidence presented in this study, including gain‑ and loss‑of‑function experiments, JNK inhibitor rescue and in vivo PPP2R3C restoration in PIL mice, the current work does not provide direct biochemical evidence, such as co‐immunoprecipitation or in vitro phosphatase assays, to demonstrate that PPP2R3C‑containing PP2A holoenzymes physically interact with or directly dephosphorylate JNK or c‑Jun. The observed regulation could be mediated through indirect mechanisms involving upstream regulators of the JNK pathway, such as MAP3K1, which has been shown to be antagonised by PPP2R3C, or through the interaction of PPP2R3C with PLCγ1 identified in our on‐going studies. Moreover, given the complexity of TCR signal transduction, PPP2R3C may exert its effects at multiple signalling nodes. Therefore, the present study serves as a foundational investigation that establishes PPP2R3C as a critical regulator of T cell activation and lupus pathogenesis. Detailed mechanistic dissection, including direct biochemical validation and structure‑function analyses, will be pursued in subsequent studies.

Our data demonstrate that PPP2R3C expression is reduced in the kidneys of SLE patients and PIL mice. In mice, PPP2R3C is expressed in podocytes, endothelial cells and mesangial cells, and its expression is downregulated in PIL mice. The superior efficacy of AAV9‑PPP2R3C, which efficiently transduces renal cells, compared to the more T‑cell‑restricted Ark313 vector, raises the possibility that PPP2R3C may exert direct renoprotective effects independent of its immunomodulatory functions. This is consistent with the observed reduction in renal p‑JNK levels upon PPP2R3C restoration. Whether PPP2R3C deficiency in renal cells directly promotes JNK‑mediated injury, and whether restoring PPP2R3C in these cells is sufficient to ameliorate nephritis, will require future studies using cell‑type‑specific gain‑ and loss‑of‑function approaches, such as podocyte‐ or endothelial‑specific conditional knockout or overexpression.

Our conclusions rely primarily on the PIL model. This model exhibits inherent inter‐animal variability, and some endpoint analyses involved only three to four mice per group. Therefore, future studies with larger cohorts and validation in genetic lupus‐prone models (e.g., MRL/lpr) are needed to strengthen the relevance of our findings to the heterogeneity of human SLE.

The present study is exploratory and hypothesis‑generating in nature. Due to the small sample sizes (typically *n* = 3–5 per group) and the exploratory design, formal corrections for multiple comparisons (e.g., Bonferroni) were not applied to most analyses. Instead, the results should be interpreted as preliminary signals requiring validation in future independent cohorts with larger sample sizes. We acknowledge that this statistical approach may increase the risk of Type I errors, but we prioritised reducing Type II errors (false negatives) to avoid obscuring potentially meaningful biological trends in this initial investigation.

## 4 MATERIALS AND METHODS

### 4.1 Human biological samples

A total of 45 SLE patients from the First Affiliated Hospital of USTC were included, all fulfilling at least four of the 2010 revised classification criteria established by the American College of Rheumatology. Peripheral blood samples were additionally acquired from 37 healthy volunteers. The demographic and clinical features of the SLE patients are summarised in Table S1. Ethical approval for this study was granted by the Hospital Ethics Review Committee (No. 2023KY‐440).

### 4.2 Mice and treatments

Six‑week‑old female BALB/c mice sourced from GemPharmatech were housed under controlled conditions. Mice were randomised into different groups. Mice were injected with AAV9‐CMV‐m‐Ppp2r3c‐3xflag‐Null, AAV9‐Null, Ark313‐m‐Ppp2r3c‐3xflag‐Null or ARK313‐Null from Hanheng Biotechnology via the tail vein to create mice with increased PPP2R3C expression. Every mouse was injected with 1 × 10^11^ virus particles, diluted to a total volume of 200 µL using PBS (pH 7.4). Following a week of receiving the AAV injection, the mice were then injected intraperitoneally .5 mL of pristane (Sigma‐Aldrich) was administered to induce a condition resembling SLE. Ten weeks later, the mice were given a second AAV9 or ARK313 injection. Ethics Committees at the First Affiliated Hospital of USTC granted approval for all animal testing conducted (approval number:2024‐N(A)‐64). The experimental protocols were followed in accordance with the Institutional Animal Care and Use Committees of the First Affiliated Hospital of USTC.

### 4.3 Cell activation

To stimulate TCR, 200 µL of complete RPMI 1640 medium was used to culture 5 × 10^5^ transfected cells for 24 h on a 48‐well plate. The wells were pre‐coated with CD3 (Biolegend) at a concentration of 5 µg/mL and soluble CD28 (Biolegend) at a concentration of 2 µg/mL. Every cell was gathered and dyed for examination using flow cytometry. Cytokine concentrations were determined by collecting supernatants after 24 h.

### 4.4 Lentiviral infection of Jurkat and human CD4+ T cell

ShRNA‐NC and shRNA‐PPP2R3C lentiviral vectors, as well as the overexpression Ctrl and overexpression PPP2R3C lentiviral vectors were purchased from Hanheng Biotechnology. Sequences of shRNA‐NC(5′ → 3′):Top strand:

GATCCGTTCTCCGAACGTGTCACGTAATTCAAGAGATTACGTGACACGTTCGGAGAATTTTTTC; Bottom strand:

AATTGAAAAAATTCTCCGAACGTGTCACGTAATCTCTTGAATTACGTGACACGTTCGGAGAACG; shRNA‐PPP2R3C(5′ → 3′): Top strand:

GATCCGCCACAATTAGATGGTCTGGAACTCGAGTTCCAGACCATCTAATTGTGGTTTTTTG; Bottom strand:

AATTCAAAAAACCACAATTAGATGGTCTGGAACTCGAGTTCCAGACCATCTAATTGTGGCG. Lentiviral infection was employed to achieve either PPP2R3C knockdown or overexpression in Jurkat cells. After a 72‑h infection period, the medium was renewed, and selection of infected Jurkat cells was carried out using 1 µg/mL puromycin. For transfection of primary T cells, CD4+ T lymphocytes were isolated and then stimulated for 3 days with 5 µg/mL anti‑CD3 antibody, 2 µg/mL anti‑CD28 antibody (Biolegend) and 100 U/mL IL‑2 (R&D). Following this, the human CD4+ T cells were infected with lentivirus at a multiplicity of infection (MOI) of 100 for 72 h. After infection, the cells were washed with RPMI 1640 medium and transferred to a culture medium lacking anti‑CD3/CD28 and IL‑2. After 2 days, the cells were re‑stimulated with 5 µg/mL anti‑CD3 antibody and 2 µg/mL anti‑CD28 antibody to assess their activation status.

### 4.5 Whole transcriptome sequencing

RNA was isolated from Jurkat cells transfected with shRNA‐GFP and shRNA‐PPP2R3C using an RNA extraction kit from Magen, following the provided guidelines (control cells *n* = 4; knockout cells *n* = 4). The whole transcriptome sequencing after PPP2R3C stable knockdown is supported by Oebiotech, and the contract number is D20221131. RNA‐sequencing data were processed with FastQC, trimmed using Trimmomatic, and further aligned to the Oryza_sativa MSU_7.0 genome using TopHat2. Differential expression analysis was conducted using EdgeR. Genes exhibiting a fold change exceeding 2 and an FDR below .01 were considered differentially expressed genes (DEGs). The FDR is defined as the *p* value adjusted by the Benjamini–Hochberg procedure. GO enrichment analysis was performed with ClusterProfiler. Volcano plots were generated by the R package ggplot2, while heatmaps were produced using pheatmap.

### 4.6 Ca^2+^ flux measurement

Transfected Jurkat and human CD4+ T cells were treated for 30 min at room temperature with 5 µM Rhod‑2/AM (Yeasen) and .05% Pluronic F127 (Beyotime). Afterwards, the cells were washed with Hanks’ balanced salt solution and incubated for an additional 30 min at room temperature to facilitate the complete de‐esterification of the AM moieties. Following dye loading, the cells were stained with anti‑CD3 (5 µg/mL) and anti‑CD28 (2 µg/mL) for 30 min. Rhod‑2 fluorescence was measured by flow cytometry at excitation 549 nm and emission 579 nm. To evaluate relative intracellular calcium flux, cells were observed for 60 s to establish a baseline. Rhod‑2 ratios were then calculated and analysed using FLOWJO, version 10.

### 4.7 Statistical analysis

GraphPad Prism was used for statistics. The Mann–Whitney *U*‐test was applied to non‑normal data and Student's *t*‑test to normal data for group comparisons. Paired *t*‑test handled paired comparisons; for multiple‐group comparisons, one‑way ANOVA with Tukey's test were used to test whether differences among the group means are statistically significant. Spearman's rank‐order test assessed correlations. Significance was defined as *p* < .05.

## AUTHOR CONTRIBUTIONS

Xuan Fang and Yi Qin: Conceptualisation, Data curation, Formal analysis, Investigation, Methodology, Writing original draft. Jinhui Tao: Conceptualisation, Project administration, Methodology. Zhou Zhou: Methodology, Data curation. Minglong Cai: Data curation. Hong Zhang: Formal analysis. Xiangpei Li: Funding acquisition, Project administration. Xiaomei Li: Funding acquisition, Project administration. Zhu Chen: Conceptualisation, Funding acquisition, Project administration, Supervision, Writing—review and editing.

## CONFLICT OF INTEREST STATEMENT

The authors declare no conflicts of interest.

## ETHICS STATEMENT

This study was approved by the Institutional Review Board of the First Affiliated Hospital of USTC (Approval No.2023KY‐440). All animal experiments were performed in accordance with the guidelines of the Institutional Animal Care and Use Committee of the First Affiliated Hospital of USTC (Approval No.2024‐N(A)‐64).

## Supporting information



Supporting Information

Supporting Information

Supporting Information

Supporting Information

Supporting Information

Supporting Information

Supporting Information

## Data Availability

All data reported in this paper will be provided by the lead contact upon request. Any additional information required to reanalyse the data reported in this paper is available from the lead contact upon request. Further information and requests for resources and reagents should be directed to and will be fulfilled by the lead contact, Zhu Chen (doczchen@ustc.edu.cn). Plasmids generated in this study are available from the lead contact without restriction.
