# Supplementary material for: PPP2R3C serves as a negative regulator associated with reduced T cell hyperactivation and renal protection in lupus
Source: Clin Transl Med. 2026 Jun 15;16(6):e70716. doi: 10.1002/ctm2.70716 (PMC13269831; doi:10.1002/ctm2.70716)
Supplement: Supplementary file 1 — Supporting Information [file CTM2-16-e70716-s004.doc]

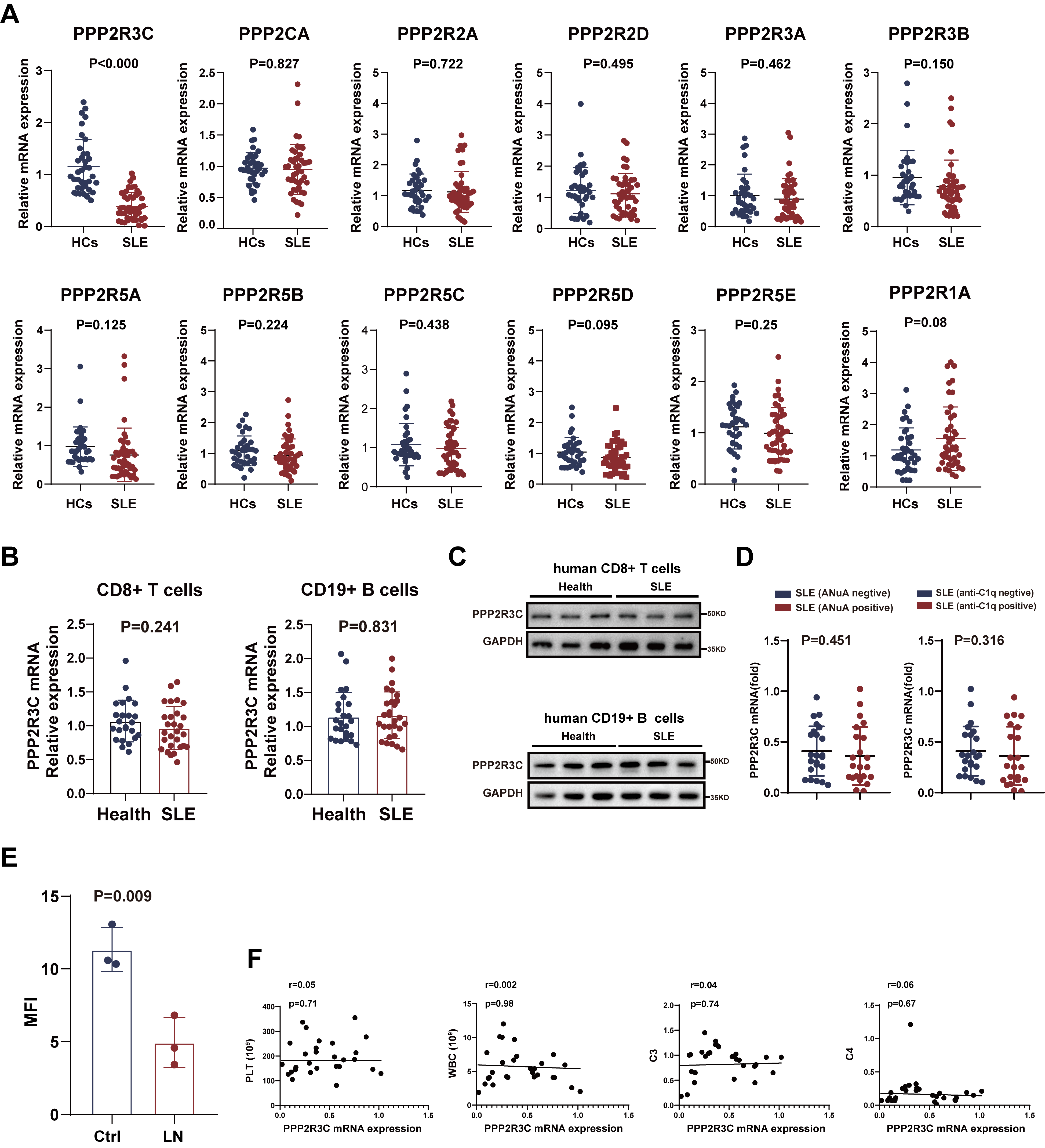


**Figure S1.** **Downregulation of PPP2R3C in SLE CD4+ T cells associated with SLE disease progression.**

**A** qRT-PCR analysis of PP2A subunits mRNA expression: PPP2R1A, PPP2CA, PPP2R2A/D, PPP2R3A/B/C, PPP2R5A/B/C/D/E mRNA in SLE and health controls CD4^+^ T cells isolated from PBMCs.

**B** The expression of PPP2R3C mRNA in SLE and health controls CD8^+^ T cells and CD19^+^ B cells.

**C** Western blot analysis of PPP2R3C protein expression in SLE and HCs CD8^+^ T cells and CD19^+^ B cells.

**D** PPP2R3C mRNA levels in CD4^+^ T cells from SLE patients stratified by seropositivity for anti-nucleosome (ANuA) and anti-C1q antibodies.

**E** Quantification of mean fluorescence intensity (MFI) of PPP2R3C in kidney biopsies from lupus nephritis patients and disease controls .

**F** Correlation analysis of PPP2R3C mRNA levels in CD4^+^ T cells from SLE with platelet count (PLT), white blood cell count (WBC), and complement C3/C4 levels.

Representative data were collected and expressed as mean±SD from three independent experiments.


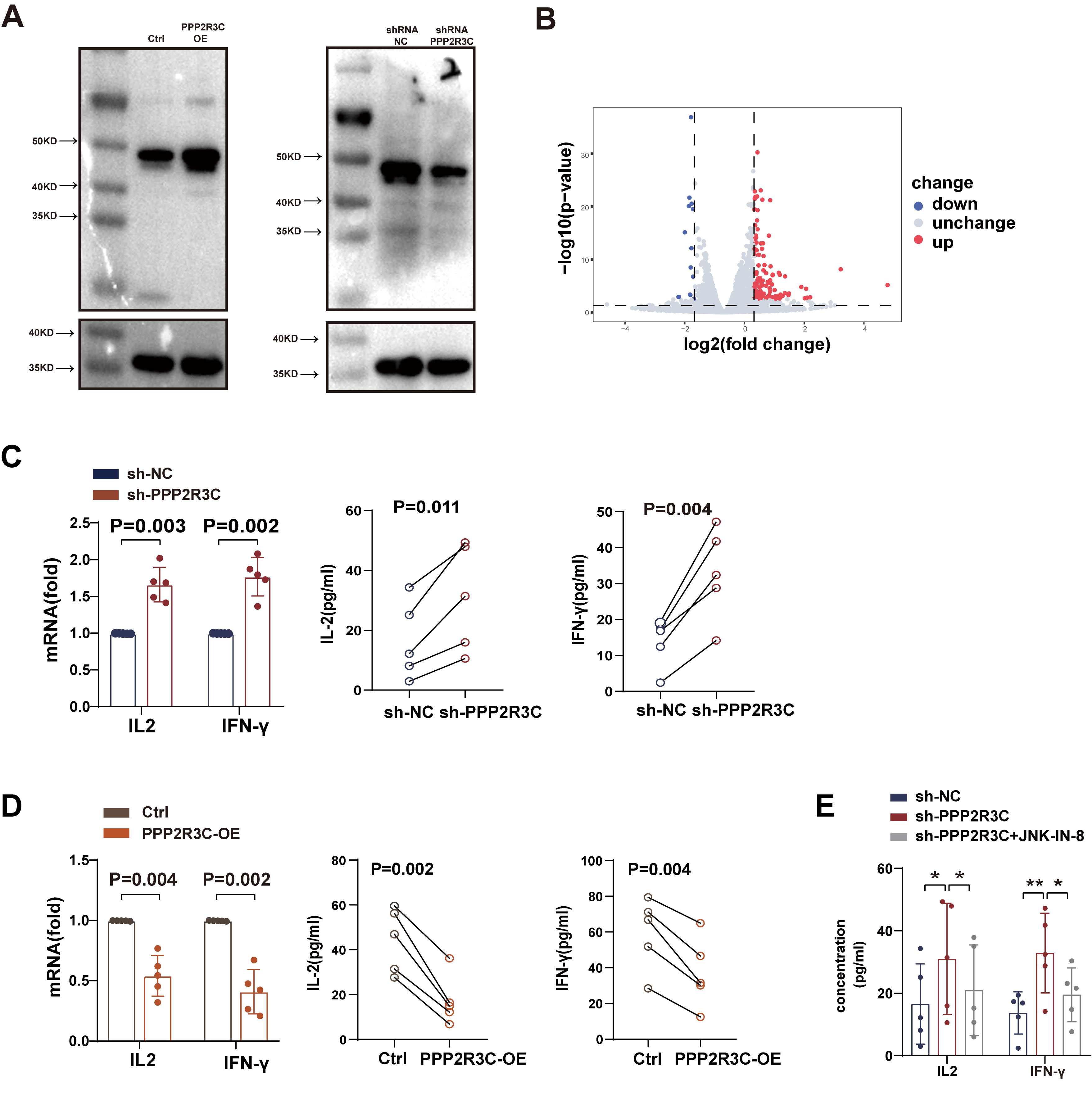


**Figure S2. PPP2R3C deficiency potentiates TCR signaling and effector functions.**

**A** The efficiency of PPP2R3C knockdown and overexpression were verified by WB.

**B** Volcano plot of RNA sequencing (RNA-seq) data from sh-NC and sh-PPP2R3C cells, highlighting 123 differentially expressed genes.

**C** IL-2 and IFN-γ mRNA levels at 3 h post-stimulation (left) and secreted protein levels at 24 h (right) in sh-NC and sh-PPP2R3C cells following anti-CD3 (5 μg/ml) and anti-CD28 antibodies (2 μg/ml).

**D** IL-2 and IFN-γ mRNA levels at 3 h post-stimulation (left) and secreted protein levels at 24 h (right) in control and PPP2R3C-OE cells following anti-CD3 (5 μg/ml) and anti-CD28 antibodies (2 μg/ml).

**E** Secreted IL-2 and IFN-γ protein levels in the supernatant of sh-PPP2R3C cells treated with or without JNK-IN-8 following anti-CD3 (5 μg/ml) and anti-CD28 antibodies (2 μg/ml) stimulation.


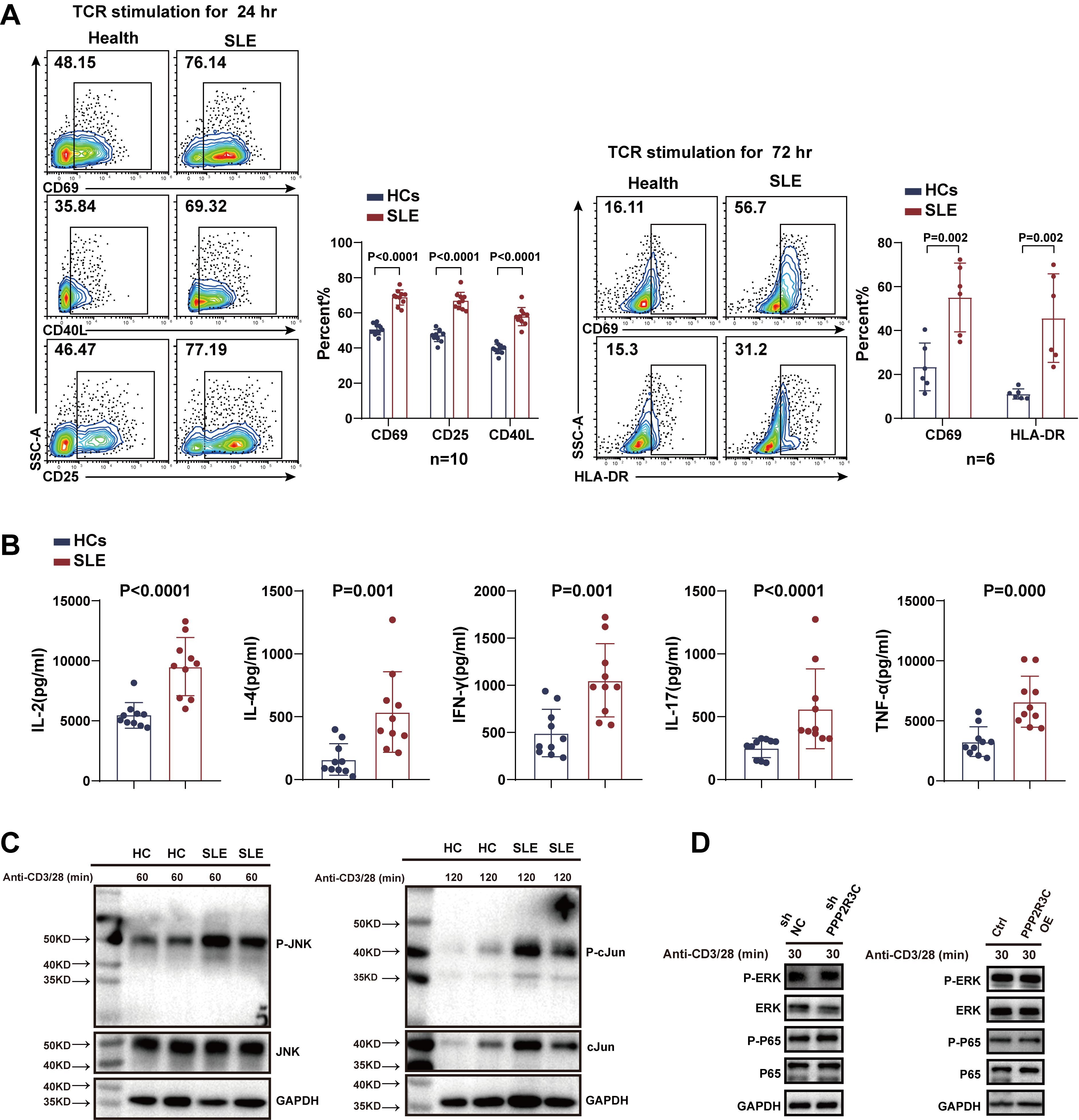


**Figure S3. PPP2R3C functions as an inhibitor of abnormal activation of CD4+ T cells in SLE patients.**

**A** Flow cytometry analysis of activation markers (CD69, CD40L, CD25, HLA-DR) on CD4^+^ T cells from HCs and patients with SLE after 24- and 72-hour TCR/CD28 stimulation .

**B** Cytokine secretion profile (IL-2, IFN-γ, TNF-α, IL-4, IL-17) in supernatants from HC and SLE CD4^+^ T cultures after 24-hour stimulation, measured by cytometric bead array (CBA).

**C** Immunoblots of phosphorylated JNK、phosphorylated c-Jun and total c-Jun in CD4⁺T cells from HCs and SLE after TCR/CD28 stimulation.

**D** Immunoblots of p65 or ERK phosphorylation upon PPP2R3C modulation after TCR/CD28 stimulation.


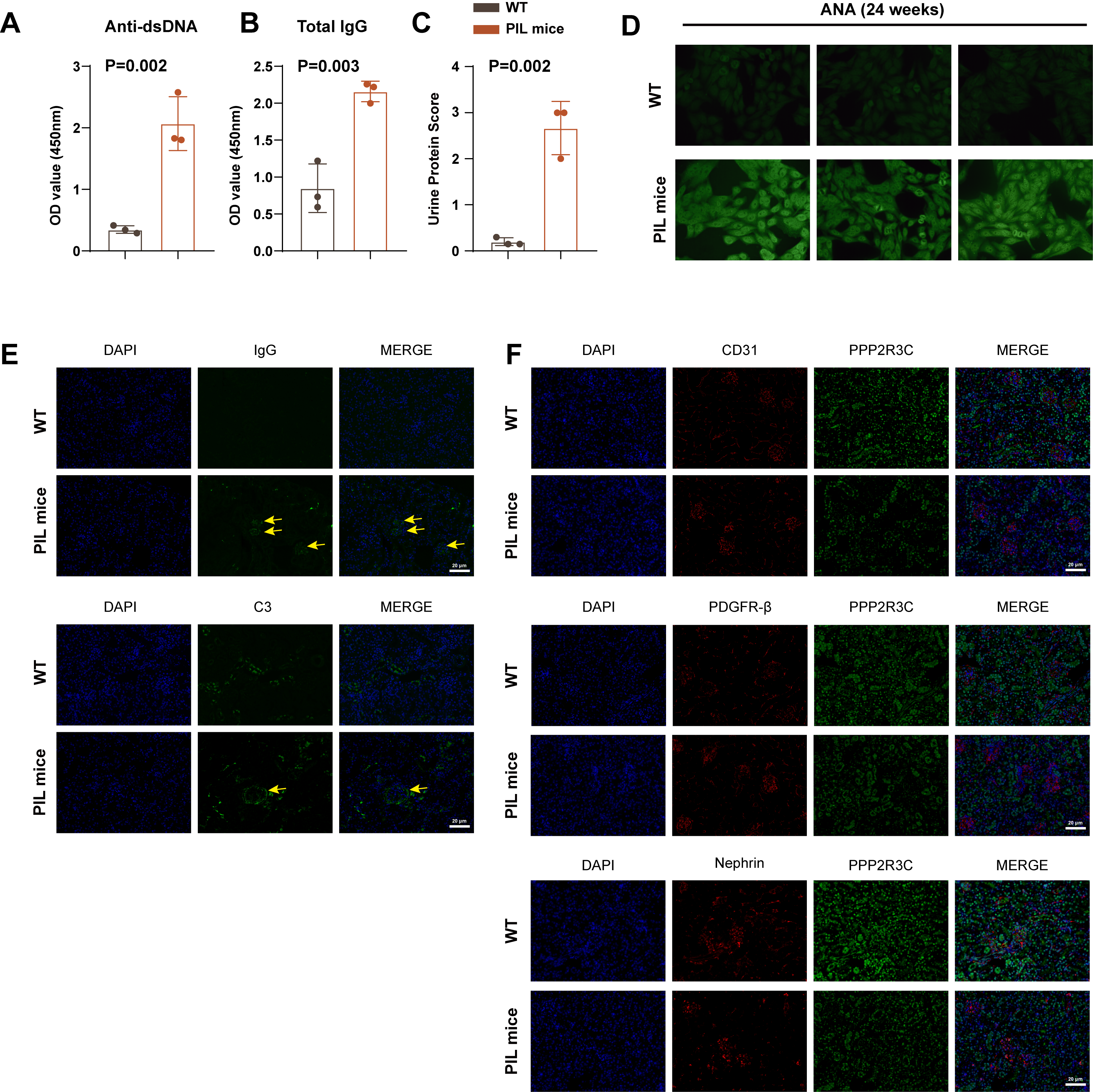


**Figure S4.** **Tissue and cell type specific dysregulation of PPP2R3C in PIL mice.**

**A** Serum levels of anti-dsDNA antibodies in WT and PIL mice at 24 weeks post-pristane injection .

**B** Serum levels of total IgG in WT and PIL mice at 24 weeks post-pristane injection.

**C** Urine albumin in WT and PIL mice.

**D** IgG/C3 immune deposits in kidneys detected via immunofluorescence (IF) in WT and PIL mice.

**F** Immunofluorescence localization of renal PPP2R3C in Nephrin⁺ podocytes, CD31⁺endothelial cells and PDGFR-β⁺mesangial cells from wild-type and PIL mice.

Representative data were collected and expressed as mean±SD from three independent experiments. *P < 0.05; **P < 0.01; ***P < 0.001; ns, not significant.


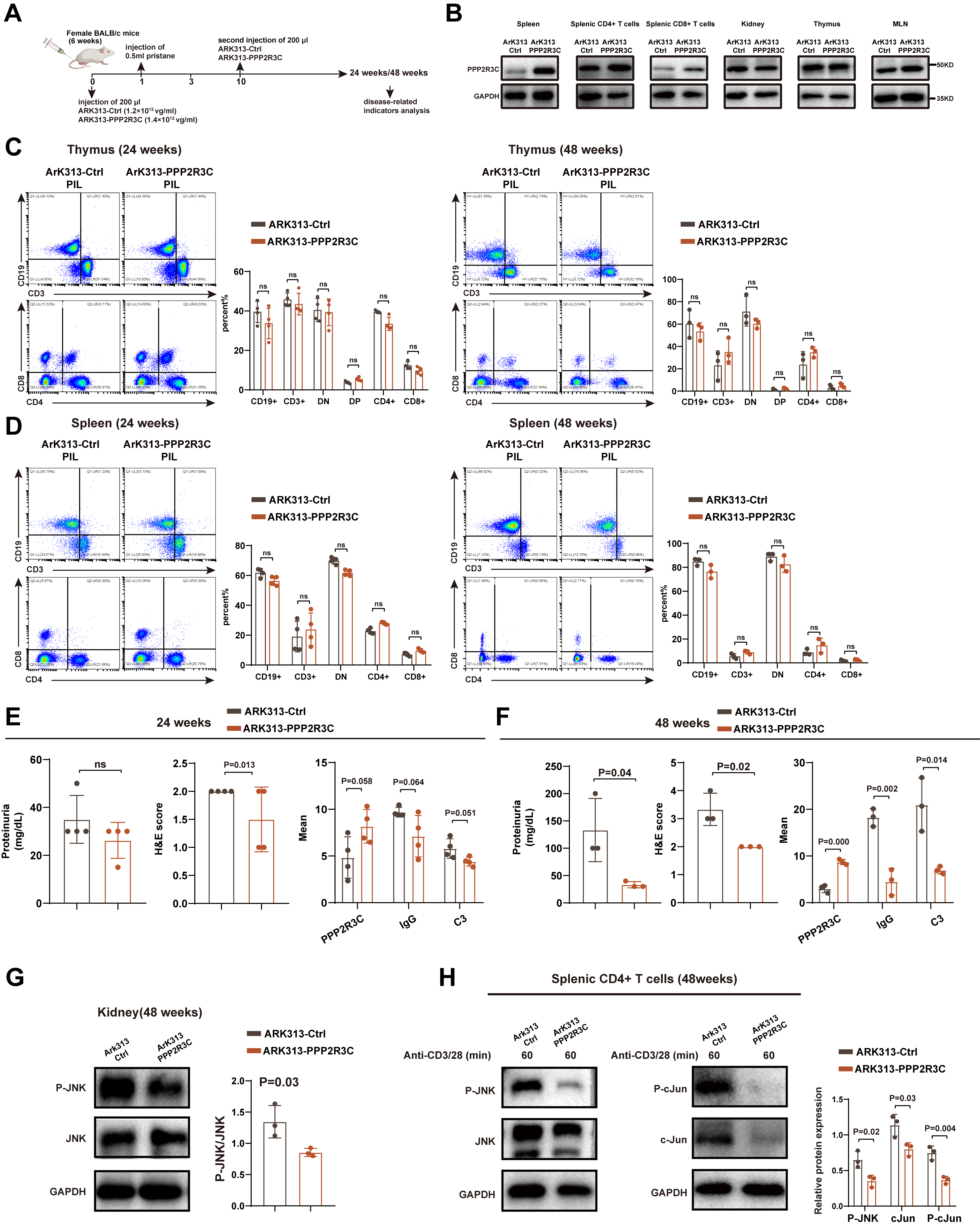


**Figure S5. T cell-targeted PPP2R3C reconstitution via Ark313 confers long-term protection in SLE.**

**A** The scheme of Ark-PPP2R3C overexpression treatment in PIL mice.

**B** PPP2R3C expression levels in the spleen, splenic CD4⁺/CD8⁺T cells, kidney, thymus, and MLN of Ark313-PPP2R3C and Ark313-ctrl PIL mice at 24 weeks.

**C** Thymic immune cell subsets in Ark313-PPP2R3C and Ark313-ctrl mice at 24 and 48 weeks.

**D** Splenic immune cell subsets in Ark313-PPP2R3C and Ark313-ctrl mice at 24 and 48 weeks.

**E** Quantitative analysis of urinary protein levels, renal H&E pathological scores, and mean fluorescence intensity (MFI) of PPP2R3C, C3, and total IgG in Ark313-PPP2R3C and Ark313-ctrl mice at 24 weeks is shown.

**F** Quantitative analysis of urinary protein levels, renal H&E pathological scores, and mean fluorescence intensity (MFI) of PPP2R3C, C3, and total IgG in Ark313-ctrl and Ark313-PPP2R3C-treated PIL mice at 48 weeks is shown.

**G** Immunoblots (left) and quantification (right) of phosphorylated JNK in kidney from Ark313-ctrl and Ark313-PPP2R3C-treated PIL mice at 48 weeks.

**H** Immunoblots (left) and quantification (right) of phosphorylated JNK、phosphorylated c-Jun and total c-Jun in splenic CD4⁺T cells from Ark313-ctrl and Ark313-PPP2R3C-treated PIL mice after TCR/CD28 stimulation at 48 weeks.

*P < 0.05; **P < 0.01; ***P < 0.001; ns, not significant.


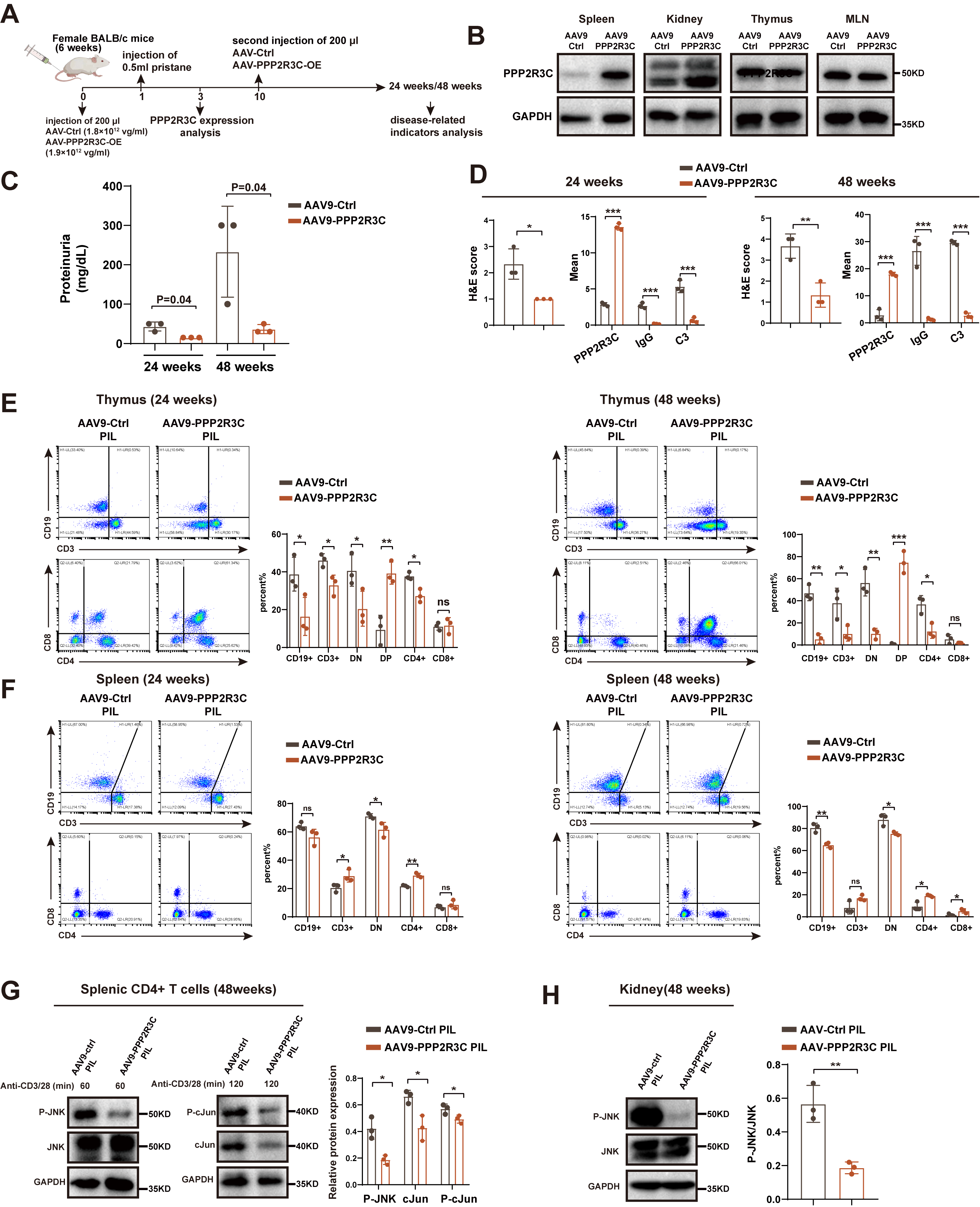


**Figure S6. Systemic PPP2R3C delivery rescued multi-organ lupus pathology in PIL mice.**

**A** The scheme of AAV9-PPP2R3C overexpression treatment in PIL mice.

**B** PPP2R3C expression levels in the spleen, kidney, thymus, and MLN of AAV9-PPP2R3C and AAV9-ctrl PIL mice at 24 weeks.

**C** Urinary protein levels in AAV9-ctrl and AAV9-PPP2R3C-treated mice at 24 and 48 weeks.

**D** **E** Thymic immune cell subsets in AAV9-ctrl and AAV9-PPP2R3C-treated mice at 24 and 48 weeks.

**F** Splenic immune cell subsets in AAV9-ctrl and AAV9-PPP2R3C-treated mice at 24 and 48 weeks.

**G** Immunoblots (left) and quantification (right) of phosphorylated JNK、phosphorylated c-Jun and total c-Jun in splenic CD4⁺T cells from AAV9-ctrl and AAV9-PPP2R3C-treated PIL mice after TCR/CD28 stimulation at 48 weeks.

**H** Immunoblots (left) and quantification (right) of phosphorylated JNK in kidney from AAV9-ctrl and AAV9-PPP2R3C-treated PIL mice at 48 weeks.

Representative data were collected and expressed as mean±SD from three independent experiments. Scale bar: 20 μm. *P < 0.05; **P < 0.01; ***P < 0.001; ns, not significant.
