# Supplementary material for: PPP2R3C serves as a negative regulator associated with reduced T cell hyperactivation and renal protection in lupus
Source: Clin Transl Med. 2026 Jun 15;16(6):e70716. doi: 10.1002/ctm2.70716 (PMC13269831; doi:10.1002/ctm2.70716)
Supplement: Supplementary file 2 — Supporting Information [file CTM2-16-e70716-s007.docx]

**Materials and methods**

**Cell culture**

Using Ficoll-Paque (TBD, Tianjin, China), PBMCs were isolated from both SLE patients and healthy controls. CD4+ T cells were subsequently purified from these PBMCs via negative selection with CD4 magnetic beads (BD Biosciences, USA). From mouse spleens, single‑cell suspensions were generated by filtering through 40 μm strainers (Falcon), and CD4+ T cells were isolated using anti‑mouse CD4 magnetic beads (BD Biosciences). Jurkat cells (ATCC, Manassas, USA) were cultured in RPMI‑1640 (GIBCO) containing 10% FBS. All cells were incubated at 37 °C in a humidified 5% CO₂ environment.

**Fluorescence quantitative real-time PCR**

qRT-PCR reverse transcription was carried out using the BioRT Master HiSensi cDNA First Strand Synthesis Kit (Bioer Technology, Hangzhou, China). Relative gene expression quantification was performed with the SYBR Green PCR Kit (Qiagen, Germany). Supplementary Table S3 contains the primer sequences. Data analysis was conducted via the ΔΔCT method and normalized to GAPDH expression levels.

**Flow cytometry**

The transfected cells were harvested and processed into a single-cell suspension by filtering through a 40-μm mesh. Surface staining involved resuspending cell pellets in 100μL of phosphate-buffered saline (PBS) with 0.5% bovine serum albumin. The mixture contained dilutions (1:100) of antibodies conjugated with fluorophores, which were then incubated at 4℃ for 30 minutes in the absence of light. Specific antibodies utilized are outlined in Table S4. The stained cells were washed with PBS and analyzed using the CytoFlex (Beckman CytoFlex, USA).

*ELISA assay.* The concentrations of serum anti-dsDNA antibodies were measured by ELISA (Alpha Diagnostic International, San Antonio, TX, USA), immunoglobulin and IFN-γ in mouse serum samples were measured by ELISA (R&D, Minneapolis, USA), All ELISA assays were performed according to the instructions provided by the manufacturers.

**ANA assay**

ANA detection was performed using indirect immunofluorescence on HEp‑2 cells (EUROIMMUN, Lübeck, Germany), and all IIF results were interpreted by a skilled laboratory technician, thereby reducing variability between observers.

**Cytokine analysis based on cytokine bead array (CBA)**

Cytokines (IL‑2, IFN‑γ, IL‑4, TNF‑α, and IL‑6) in supernatants of T cells activated via TCR were quantified using the BD™ CBA Human Th1/Th2 Cytokine Kit II (BD, USA). This bead‑based flow cytometric assay detects soluble analytes using beads of defined size and fluorescence.

**Western blot analysis**

The process of breaking down cells was carried out by utilizing RIPA buffer along with protease and phosphatase inhibitors (EpiZyme, Shanghai, China) for a duration of 15 minutes on ice. This was then followed by heating the entire cell lysate in SDS sample buffer for 10 minutes. Afterwards, the entire protein content was isolated through 10% SDS-PAGE (EpiZyme, Shanghai, China). Next, a PVDF membrane (Millipore, USA) was used. After blocking with Protein Free Rapid Blocking Buffer (EpiZyme, Shanghai, China) for 20 min at room temperature, the membrane was incubated overnight at 4 °C on a shaker in a solution containing antibodies diluted with antibody diluent.(EpiZyme, Shanghai, China), with primary antibodies diluted at a ratio of 1:1000 (refer to Table S5 for specific antibody details).

**Histochemistry and immunohistochemistry**

The kidneys were extracted and immersed in 10% neutral buffered formalin for 48 hours for histological examination. Afterward, they were trimmed, dehydrated, and encased in paraffin wax. Samples with a thickness of 3-5 µm were created, placed on glass slides, treated with xylene to remove paraffin, and then rehydrated using different concentrations of alcohol. Finally, they were stained with either H&E. The evaluation of the slides were done by two experienced pathologists in a blinded fashion.

The kidneys were promptly frozen in OCT after extraction to identify glomerular immune complexes using immunofluorescence. Sections of the cryostat (4 µm) were treated with the specified antibodies AF488 goat anti-mouse IgG (Abcam, USA), Rabbit anti-mouse C3 (Proteintech, China) and then FITC labeled goat anti-rabbit IgG (Abbkine, China). Nuclei were visualized by incubating embryos in DAPI. Photos were taken with a ZEISS microscope, and Analysis was conducted using ImageJ open-source software. Glomeruli-containing regions were manually delineated as regions of interest, with background exclusion achieved through intensity thresholding; the proportion of area above the threshold in these regions was quantified in three separate fields of view per sample and reported as a percentage of the total glomerular area.
