# Supplementary material for: PPP2R3C serves as a negative regulator associated with reduced T cell hyperactivation and renal protection in lupus
Source: Clin Transl Med. 2026 Jun 15;16(6):e70716. doi: 10.1002/ctm2.70716 (PMC13269831; doi:10.1002/ctm2.70716)
Supplement: Supplementary file 3 — Supporting Information [file CTM2-16-e70716-s003.doc]

**Table S1. Demographic and clinical characteristics of SLE patients.**

|  | SLE | HCs |
| --- | --- | --- |
| Gender (female/male, n) | 43/2 | 34/3 |
| Age (mean ± SD, Year) | 38.38±10.04 | 37.11±9.08 |
| SLEDAI-2K (mean ± SD) | 6.04±3.82 |  |
| Duration of diagnosis(months) | 57.95±60.52 |  |
| **Laboratory Manifestations** |  |  |
| Anti-dsDNA + (n, %) | 31, (68.9) |  |
| Anti-nucleosome + (n, %) | 25, (55.6) |  |
| Anti-nuclear + (n, %) | 45, (100) |  |
| Anti-U1RNP + (n, %) | 22, (48.9) |  |
| Anti-Sm + (n, %) | 12, (26.7) |  |
| Anti-C1q + (n, %) | 18, (40) |  |
| **Immunosuppressive therapy** |  |  |
| Prednisone or methylprednisolone（n, %） | 36,(80) |  |
| Hydroxychloroquine (n, %) | 36,(80) |  |
| Cyclophosphamide (n, %) | 3,(3.4) |  |
| Mycophenolate mofetil (n, %) | 7,(15.6) |  |
| Cyclosporin A (n, %) | 2,(4) |  |
| Leflunomide (n, %) | 4,(8.9) |  |
| Tacrolimus (n, %) | 1,(2) |  |
