# Supplementary material for: PPP2R3C serves as a negative regulator associated with reduced T cell hyperactivation and renal protection in lupus
Source: Clin Transl Med. 2026 Jun 15;16(6):e70716. doi: 10.1002/ctm2.70716 (PMC13269831; doi:10.1002/ctm2.70716)
Supplement: Supplementary file 4 — Supporting Information [file CTM2-16-e70716-s006.doc]

**Table S2. Demographic of Lupus Nephritis patients**

| **Sample** | **Gender** | **Age (year)** | **Biopsy type** | **Diagnosis** |
| --- | --- | --- | --- | --- |
| Control patient1 | male | 37 | needle | IgA nephropathy |
| Control patient2 | male | 29 | needle | diffuse mesangial cell proliferation （mild） |
| Control patient3 | male | 48 | needle | Obesity-associated glomerulomegaly nephropathy |
| Patient 1. | female | 53 | needle | Class IV-G lupus nephritis |
| Patient 2. | female | 34 | needle | Class V lupus nephritis |
| Patient 3. | fmale | 55 | needle | Class III lupus nephritis |
