# Supplementary material for: PPP2R3C serves as a negative regulator associated with reduced T cell hyperactivation and renal protection in lupus
Source: Clin Transl Med. 2026 Jun 15;16(6):e70716. doi: 10.1002/ctm2.70716 (PMC13269831; doi:10.1002/ctm2.70716)
Supplement: Supplementary file 5 — Supporting Information [file CTM2-16-e70716-s002.doc]

**Table S3. Primer sequences for indicated genes**

| All primers are listed in 5' to 3' sequence | | |
| --- | --- | --- |
|  | Forward | Reverse |
| Human GAPDH | ACCACCCTGTTGCTGTAGCCAA | GTCTCCTCTGACTTCAACAGCG |
| Human PPP2R3C | ACGTTGCCACAATTAGATGGT | GGCCATAAACTCTTAGGGCAGAA |
| Human PPP2R2D | AAGACTTCGAGACCCATTTAGGA | CGTGGACTCGCTTCTACCATA |
| Human PPP2R5C | TGGCACTTCTCAAATACTGGC | CGCTGCGTTGTCACTGATTAAA |
| Human PPP2R1A | TAGACGAACTCCGCAATGAGG | CACCAGGGTAGTGAAGGTTCC |
| Human PPP2CA | GGTGGTCTCTCGCCATCTATAG | CTGGATCTGACCACAGCAAGTC |
| Human PPP2R2A | GCAACAGGAGATAAAGGTGGTAG | TGGTTCATGGCTCTGGAAGGTG |
| Human PPP2R5B | CTGAAGACGGAGCACAAGCAGT | TCAGAGTGGCATCCTTCTCCAG |
| Human  PPP2R3B | ACTACCTGGTGCAGGAGGACTT | GTGATGTAGCGCGAGTGGAACT |
| Human  PPP2R3A | CTATGAGGAGCAGTGTGAACGG | GATGTGAGCCATTCTGCACCTC |
| Human  PPP2R5E | GACAGAAGAGGTCGCAAAGTTCC | AGGAACAGTTCAGGCTGCTCTG |
| Human  PPP2R5D | TACGAGACGGAGCATCACAACG | GGAAGTAGGACACGGATGAGGA |
| Human IL2 | TCCTGTCTTGCATTGCACTAAG | CATCCTGGTGAGTTTGGGATTC |
| Human IFN-γ | TCGGTAACTGACTTGAATGTCCA | TCGCTTCCCTGTTTTAGCTGC |
| Mouse- PPP2R3C | CTTCGGGAATCAGACCTGGAG | GCAGTGCAGACATAGAAGGAGT |
| Mouse-GAPDH | AGGTCGGTGTGAACGGATTTG | GGGGTCGTTGATGGCAACA |
