# Supplementary material for: PPP2R3C serves as a negative regulator associated with reduced T cell hyperactivation and renal protection in lupus
Source: Clin Transl Med. 2026 Jun 15;16(6):e70716. doi: 10.1002/ctm2.70716 (PMC13269831; doi:10.1002/ctm2.70716)
Supplement: Supplementary file 6 — Supporting Information [file CTM2-16-e70716-s001.doc]

**Table S4. Antibodies used for flow cytometry**

|  | clone | company | Cat.No |
| --- | --- | --- | --- |
| anti-human CD69-Percp/Cy5.5 | FN50 | biolegend | 310926 |
| anti-human CD25-APC | BC96 | biolegend | 302610 |
| anti-human CD40L-AF700 | 24-31 | biolegend | 310846 |
| anti-human CD4-APC | RPA-T4 | biolegend | 561841 |
| anti-human HLA-DR-BV510 | L243 | biolegend | 307645 |
| Zombie NIR™ Fixable Viability Kit |  | biolegend | 423106 |
| anti-mouse CD25-APC | PC61 | biolegend | 102012 |
| anti-mouse CD4-PE | RM4-5 | BD Biosciencee | 553048 |
| anti-mouse CD40L-APC | SA047C3 | biolegend | 157009 |
| anti-mouse CD69-FITC | H1.2F3 | biolegend | 104505 |
| anti-mouse CD4-FITC | GK1.5 | biolegend | 100406 |
| anti-mouse CD44-APC | IM7 | biolegend | 103012 |
| anti-mouse CD62L APC/R700 | MEL-14 | BD Bioscience | 565159 |
| anti-mouse CD8-APC/Cy7 | 53-6.7 | biolegend | 100714 |
| anti-mouse CD19-Pacific Blue | 6D5 | biolegend | 115523 |
| anti-mouse CD3e-PerCP/Cy5.5 | 145-2C11 | BD Bioscience | 551163 |
