# Supplementary material for: PPP2R3C serves as a negative regulator associated with reduced T cell hyperactivation and renal protection in lupus
Source: Clin Transl Med. 2026 Jun 15;16(6):e70716. doi: 10.1002/ctm2.70716 (PMC13269831; doi:10.1002/ctm2.70716)
Supplement: Supplementary file 7 — Supporting Information [file CTM2-16-e70716-s005.doc]

**Table S5. Antibodies used for Western-blot**

| anti | Company | Cat.No. |
| --- | --- | --- |
| GAPDH | EpiZyme | LF206 |
| PPP2R3C | Abcam | ab230347 |
| JNK | Cell Signaling Technology | 9252S |
| P-JNK | Cell Signaling Technology | 9255S |
| ERK | Cell Signaling Technology | 4695T |
| P-ERK | Cell Signaling Technology | 4370T |
| P65 | Cell Signaling Technology | 8242T |
| P-P65 | Cell Signaling Technology | 3033T |
| cjun | Cell Signaling Technology | 9165S |
| P-cjun | Cell Signaling Technology | 3270S |
